# Structural Properties of Synaptic Transmission and Temporal Dynamics at Excitatory Layer 5B Synapses in the Adult Rat Somatosensory Cortex

**DOI:** 10.3389/fnsyn.2018.00024

**Published:** 2018-07-30

**Authors:** Astrid Rollenhagen, Ora Ohana, Kurt Sätzler, Claus C. Hilgetag, Dietmar Kuhl, Joachim H. R. Lübke

**Affiliations:** ^1^Institute of Neuroscience and Medicine INM-2, INM-10, Research Centre Jülich GmbH, Jülich, Germany; ^2^Institute of Molecular and Cellular Cognition, Center for Molecular Neurobiology, University Medical Center Hamburg-Eppendorf, Hamburg, Germany; ^3^School of Biomedical Sciences, University of Ulster, Coleraine, United Kingdom; ^4^Institute of Computational Neuroscience, University Medical Center Hamburg-Eppendorf, Hamburg, Germany; ^5^Department of Psychiatry, Psychotherapy and Psychosomatics, RWTH Medical University Aachen, Aachen, Germany; ^6^JARA-Brain Medicine, Aachen, Germany

**Keywords:** barrel cortex, layer 5B synapses, paired recordings, synaptic transmission, quantal analysis, electron microscopy, 3D-reconstructions, quantitative 3D-models of synaptic boutons

## Abstract

Cortical computations rely on functionally diverse and highly dynamic synapses. How their structural composition affects synaptic transmission and plasticity and whether they support functional diversity remains rather unclear. Here, synaptic boutons on layer 5B (L5B) pyramidal neurons in the adult rat barrel cortex were investigated. Simultaneous patch-clamp recordings from synaptically connected L5B pyramidal neurons revealed great heterogeneity in amplitudes, coefficients of variation (CVs), and failures (F%) of EPSPs. Quantal analysis indicated multivesicular release as a likely source of this variability. Trains of EPSPs decayed with fast and slow time constants, presumably representing release from small readily releasable (RRP; 5.40 ± 1.24 synaptic vesicles) and large recycling (RP; 74 ± 21 synaptic vesicles) pools that were independent and highly variable at individual synaptic contacts (RRP range 1.2–12.8 synaptic vesicles; RP range 3.4–204 synaptic vesicles). Most presynaptic boutons (~85%) had a single, often perforated active zone (AZ) with a ~2 to 5-fold larger pre- (0.29 ± 0.19 μm^2^) and postsynaptic density (0.31 ± 0.21 μm^2^) when compared with even larger CNS synaptic boutons. They contained 200–3400 vesicles (mean ~800). At the AZ, ~4 and ~12 vesicles were located within a perimeter of 10 and 20 nm, reflecting docked and readily releasable vesicles of a putative RRP. Vesicles (~160) at 60–200 nm constituting the structural estimate of the presumed RP were ~2-fold larger than our functional estimate of the RP although both with a high variability. The remaining constituted a presumed large resting pool. Multivariate analysis revealed two clusters of L5B synaptic boutons distinguished by the size of their resting pool. Our functional and ultrastructural analyses closely link stationary properties, temporal dynamics and endurance of synaptic transmission to vesicular content and distribution within the presynaptic boutons suggesting that functional diversity of L5B synapses is enhanced by their structural heterogeneity.

## Introduction

Synapses between different neurons in the brain are tuned to support computations specific to the neural networks in which they are embedded. This tuning is achieved, in part, by adaptations and specialization of their structural composition. Hence, detailed knowledge of synaptic structure is vital for understanding the mechanisms underlying synaptic function. Meanwhile several comprehensive studies have described structure-function relationships in peripheral (Dawson-Scully et al., 2007; Ehmann et al., 2014; reviewed by Denker et al., 2009), sensory (reviewed by Wichmann and Moser, 2015), brain stem (Rowland et al., 2000; Sätzler et al., 2002; reviewed by Borst and van Hoeve, 2012) and hippocampal (Harris and Sultan, 1995; Schikorski and Stevens, 1997; Rollenhagen et al., 2007; Branco et al., 2010; reviewed by Bischofberger et al., 2006; Rollenhagen and Lübke, 2010; Harris and Weinberg, 2012), and cerebellar synapses (Xu-Friedman et al., 2001; Xu-Friedman and Regehr, 2003). These studies demonstrated that structural subelements, in particular the number, size and organization of active zones (AZs) and that of the pools of synaptic vesicles play a pivotal role in synaptic transmission, but are organized in unique ways to foster functional specializations. For example, high efficacy and fidelity of synaptic transmission at brain stem synapses is guaranteed by a parallel assembly of hundreds of small variable release sites (Schneggenburger et al., 1999; Sätzler et al., 2002; reviewed by Borst and van Hoeve, 2012), whereas in hippocampal CA1 synapses containing only 1–2 release sites, increased transmitter release probability (*P*_*r*_) and efficacy are correlated with larger AZs and PSDs (Matz et al., 2010; Holderith et al., 2012). Synaptic strength and reliability also depend on the mode of release: uni- vs. multivesicular, synchronous or asynchronous (reviewed by Neher, 2015; Rudolph et al., 2015; Chamberland and Tóth, 2016), although their correlation with specific AZ morphology, remains unclear. During ongoing activity, temporal dynamics and persistence of release depend on the continuous availability of vesicles primed for release. Based on this availability, readily releasable (RRP), recycling (RP), and resting pools have been identified. In a few synapses these functional pools map onto the geometrical distance of their vesicles from the AZ (Kuromi and Kidokoro, 1998), but in the majority only a partial correlation exists between location and function (reviewed by Denker and Rizzoli, 2010; Alabi and Tsien, 2012; Fowler and Staras, 2015; Chamberland and Tóth, 2016). In all synapses, vesicle numbers and their geometrical distance to the AZ provide important boundaries for their utilization. The role of the resting pool remains enigmatic as it seldom appears to be modified, however, some evidence to its involvement in strong synaptic activation (Kuromi and Kidokoro, 2000; Denker et al., 2009) and plasticity (Wang et al., 2016) has been acquired. Whether a resting pool exists in all synapses and under which conditions it might be utilized, also remains unresolved.

Cortical synapses are the most abundant in the brain and participate in various computations underlying perception, executive control, learning and memory. Multiple patch-clamp recordings *in-vitro* and *in-vivo* revealed a great diversity among cortical synapses with respect to their size, reliability, and temporal dynamics (reviewed by Lübke and Feldmeyer, 2007; Feldmeyer et al., 2013). However, comprehensive studies of synaptic structure and its relation to function are still very rare for cortical synapses (Rollenhagen et al., 2015; Bopp et al., 2017; Hsu et al., 2017). Consequently, the mechanisms by which their function and diversity are generated are still unresolved. Major difficulties in addressing these questions are imposed by the small size of cortical synapses, their inaccessibility for direct measurements and the heterogeneity of their pre- and postsynaptic neurons.

To fill this gap paired recordings, quantal analysis, high-end fine-scale electron microscopy (EM) and quantitative 3D-volume reconstructions of individual synaptic boutons in L5B were performed. To reduce ambiguity due to neural and synaptic heterogeneity, we targeted L5B synapses residing on basal dendrites only.

Large L5B thick-tufted pyramidal neurons in rodent somatosensory cortex are interconnected via single-axon synaptic contacts located predominantly on their basal dendrites (Markram, 1997; Markram et al., 1997a,b). At early stages of development, these synapses exhibit relatively large and invariable EPSPs (also referred to as unitary EPSPs, uEPSPs) and strong frequency dependent depression (Markram, 1997; Markram et al., 1997a,b, 1998; Ohana and Sakmann, 1998; Frick et al., 2007, 2008). However, later in development (postnatal week 3–5), L5B-L5B synapses display small and unreliable EPSPs that remain constant or facilitate during trains of action potentials (Reyes and Sakmann, 1999; Williams and Atkinson, 2007; Hardingham et al., 2010; Kerr et al., 2013). In addition, a consistent finding is that the CV and *P*_*r*_ vary greatly between L5B synapses in young-adult neocortex. A possible, but yet unexplored source of this variance is that vesicular content, distribution and supply rates differ among these synapses or even between individual synapses in a given connection. We thus performed recordings and analyses of L5B synapses focused on estimating functional vesicular pools involved in stationary and temporally modulated release.

This was followed by quantitative 3D-reconstructions of synaptic boutons (equivalent to the recorded synapses) that lead to realistic values of synaptic densities, size of boutons, AZs, number of vesicles per bouton, and their precise geometrical distribution, mitochondrial occupancy, and astrocytic coverage.

Our findings suggest that structural heterogeneity underlies and explains functional diversity, which could expand the computational range and promote fast transitions between transmission states at individual synapses. Moreover, our data provide values and constraints essential for constructing realistic 3D synaptic models and for numerical (MonteCarlo) simulations of various aspects of transmitter release. Through comparison of electrophysiological and morphological measurements, the release modus and vesicular pools dominating at this synapse were defined.

## Materials and methods

### Experimental procedures

All experiments were approved by the Animal Research Committee of the Research Centre Jülich GmbH, the local authorities of the City of Hamburg, and complied with the guidelines laid out in the EU directive regarding the protection of animals used for experimental and scientific purposes (2004/23/EC).

#### Brain slices and solutions

Wistar rats (30–35 days old) were anesthetized with isoflurane, decapitated and slices (thickness 300–350 μm) from the somatosensory area were prepared in ice-cold oxygenated (95% O_2_, 5% CO_2_) recording-ACSF or slicing-ACSF using a vibrating microslicer (Mikrom HM 650V, Thermo Scientific, Walldorf, Germany). Sagittal slices (300–350 μm) were cut at a 10° angle from the midline, incubated for 30 min in recording-ACSF at 36°C and subsequently transferred to room temperature until recording. Slicing-ACSF contained (in mM): 87 NaCl, 75 sucrose, 26 NaHCO_3_, 2.5 KCl, 1 NaH_2_PO_4_, 7 MgCl_2_, 0.5 CaCl_2_, 10 glucose. Recording-ACSF contained (in mM): 125 NaCl, 25 NaHCO_3_, 2.5 KCl, 1.25 NaH_2_PO_4_, 1 MgCl_2_, 2 CaCl_2_, and 25 glucose. In some experiments CaCl_2_ was increased to 2.5 mM and MgCl_2_ to 1.3 mM. For measurements of mEPSPs TTX (1 μM) and Bicuculine (30 μM) were added to the ACSF.

#### Cell identification and electrophysiology

Recordings were made in a submerged chamber continuously perfused (3 ml/min) with oxygenated ACSF maintained at 34–37°C. Neurons were visualized with IR-DIC microscopy using an Olympus BX51WI microscope equipped with x60 water-immersion objective (Olympus, Hamburg, Germany). Recordings were made simultaneously from two to four synaptically coupled L5B thick-tufted pyramidal neurons typically located within 20–150 μm from each other. Pipettes (4–6 MΩ) were pulled from borosilicate glass and filled with a recording solution containing (in mM): 105 potassium gluconate, 30 KCl, 10 Hepes, 10 phosphocreatine-Na_4_ ATP-Mg, and 0.3 guanosine triphosphate, osmolarity was adjusted to 280–290 mOsm and biocytin (0.1–0.5 mg/ml, Sigma, Munich, Germany) or horseradish peroxidase (HRP; 0.1–0.5 mg/ml, Sigma, Munich, Germany) was included in the recording solution for subsequent identification and morphological analysis of the recorded neurons. Pipette solution was mixed thoroughly and filtered again after addition of biocytin but not HRP. HRP-containing solution was backfilled in pipettes containing HRP-free solution in the tip (~3 μl). Once added, patching had to be completed within 60 s, since HRP prevented the formation of a Gigaseal.

Somatic whole-cell patch-clamp recordings were made in the current-clamp mode (Multiclamp-700a/b amplifier, Molecular Devices, Sunnyvale, CA, USA). Data acquisition was done online through an A-D converter (Digidata 1322/1422, Molecular Devices, Sunnyvale, CA, USA) at a sampling rate of 10 kHz and filtered at 3 kHz. Liquid-junction potential was corrected via the designated algorithm in Multiclamp 700 amplifiers, the access resistance continuously monitored and bridge potential compensated. Typical access resistance under these conditions was between 6 and 20 MΩ. Data were collected and visualized using the pClamp10 software (Molecular Devices, Sunnyvale, CA, USA), exported and analyzed offline, using Clampfit10 and custom written routines in IGOR Pro 6.3 (WaveMetrics Inc., Lake Oswego, OR, USA).

#### EPSP measurements and analysis

EPSPs were measured from single-axon connections, which are elsewhere referred to as unitary EPSPs (uEPSPs). For simplicity and easier comparison to relevant literature, we use here the term EPSP. EPSP amplitudes were analyzed from series of consecutive (30–180) sweeps repeated every 8–20 s. A single presynaptic AP was evoked per sweep, or in a few cases 3 at a 10 Hz frequency. In all cases parameters of the first EPSP were used for quantal estimates. All sweeps were aligned to the AP peak and an averaged-EPSP was constructed. A time window for EPSP detection was defined between the AP peak and 67% decay of the averaged-EPSP (typically 5–8 ms). An identically sized time window was placed just prior to the stimulus, to measure the baseline amplitude. Within these windows, the maximal amplitude was automatically detected and averaged over 10 sampling points. The EPSP amplitude was calculated by subtracting the mean baseline from the peak EPSP. Failures were detected manually by comparing individual sweeps to the averaged-EPSP and defined as absence of any EPSP-like event within the EPSP time window. CV was noise-subtracted and measured as previously described (Ohana et al., 2012). *P*_*r*_ was calculated from the following equation:

$$\begin{array}{l} \frac{1}{CV^{2}}=\frac{N\times P}{1-p} \end{array}$$

and q as:

$$\begin{array}{l} q=\frac{\bar{EPSP}}{N\times P} \end{array}$$

The number of release sites N was assumed to be the same as the number of synaptic contacts identified from the 3D-reconstructions, or when unavailable, a mean of *N* = 3 was used which is close to the mean number (3.5 ± 0.5) from the Neurolucida reconstructions.

#### Measurements and analysis of mEPSPs

Spontaneous mEPSPs were measured in current-clamp under the same conditions as the EPSPs. To isolate spontaneous glutamatergic release, biccuculine (10 μM) and TTX (1 μM) were added to the ACSF. Continuous 5–10 min long recordings were filtered at 1 kHz and 100–500 mEPSPs per neuron were detected and analyzed based on threshold-crossing and visual inspection (MiniAnalysis software, Synaptosoft, Decatur, GA, USA).

#### Quantal binomial model fits of EPSP amplitude histograms

The algorithm used was developed and explained in detail by Hardingham et al. (2006, 2010) and made available by Jenny Read at www.jennyreadresearch.com. EPSPs were selected and analyzed as described above. Histograms of the background noise were constructed and fitted with a Gaussian function for each individual experiment, and the SD was implemented in the model. The model was allowed to search for an optimal N ranging from 1 to 20. The adequacy tests included a comparison to the observed failure rate.

#### Functional pools estimates from trains of EPSPs

Repetitive trains (20–50, inter-train intervals 20 s) of 10–50 APs (intra-train frequency 10–100 Hz) were elicited in the presynaptic neuron. Evoked trains of EPSPs were aligned to the peak of the first AP in the train, baseline subtracted and averaged. Mean EPSP amplitudes were measured from the averaged trace. Exponential fits were made in IGOR Pro (WaveMetrics Inc., Lake Oswego, OR 97035, USA) using the double-exponential function, to capture the rapid- and slow-decay constants of the EPSP amplitude. The exponential function was forced to reach an end-value of zero since a full depletion was not achieved in all synapses but was required for a total pool size estimate. The area underneath each exponential was calculated and divided by the mean mEPSP amplitude to obtain an estimate of the number of vesicles released during each phase (=exponent) of the train.

Physiological data points in the figures represent means of each connection. Data are presented as mean ± SD. Linear correlations were tested in IGOR Pro. *T*-tests were performed with a significance level of *p* < 0.05.

After recording, slices containing the biocytin- or HRP-filled neurons were immersion-fixed with 4% paraformaldehyde and 0.1–0.5% glutaraldehyde diluted in 0.1 M phosphate buffer (PB, pH 7.4) overnight and then further processed for EM as described in detail below for the perfusion-fixed material.

### Structural investigation and analysis

#### Morphological reconstructions of biocytin- and HRP-filled synaptically coupled neurons

For light- and EM analysis slices containing biocytin- and HRP-filled neurons were processed using a modified protocol previously described (Lübke et al., 2000). Finally, slices were reacted using 3,3-diaminobenzidine (DAB) as a chromogen under light microscopic control until dendritic and axonal arborizations of the pre- and postsynaptic neurons were clearly visible. Slices were then briefly post-fixed in 0.5% OsO_4_ (30 min) diluted in 0.1 M phosphate buffer (PB; pH 7.4). After thorough washing steps in 0.1 M PB they were conventionally embedded for EM (see below).

Biocytin- and HRP-labeled pairs of neurons were reconstructed with the Neurolucida software (MicroBrightfield, Colchester, VT, USA) using an Olympus BX50 microscope (Olympus, Hamburg, Germany) at a final magnification of × 780 or × 1200. Synaptically coupled pairs of neurons were examined to identify the number, location, and geometric distance of putative synaptic contacts. Potential synaptic contacts were identified as close appositions of a synaptic bouton and the postsynaptic dendrite in the same focal plane at a final magnification of × 1200. For all data, means ± SDs are given. Data were not corrected for shrinkage.

#### Fixation and tissue processing for 3D-reconstructions of excitatory synaptic boutons in L5B

**Figure 1:**
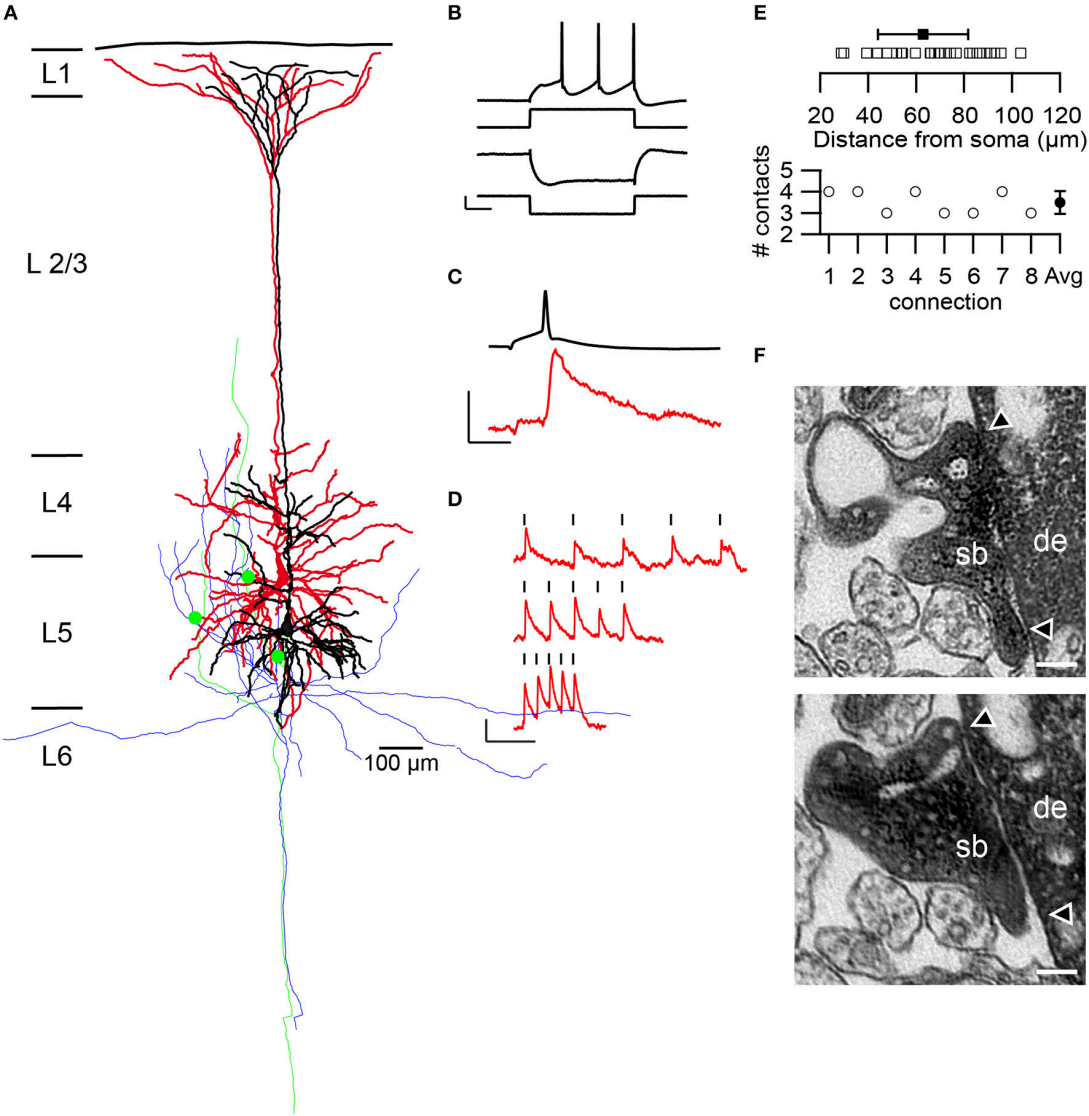
Structural and functional characteristics of L5B thick-tufted pyramidal neurons and synapses. **(A)** Neurolucida reconstruction of a synaptically coupled pair of L5B thick-tufted pyramidal neurons filled with HRP. The dendritic tree of the presynaptic neuron is given in black and that of the postsynaptic neuron in red, the axonal arborizations in green and blue, respectively. Three synaptic contacts (green dots) were established at different locations on the basal dendritic tree of the postsynaptic neuron. **(B–D)** Example recordings from the pair shown in **(A)**. Pre- and postsynaptic membrane potentials are depicted in black and red, respectively. **(B)** L5B thick-tufted pyramidal neurons typically exhibited a sagging membrane potential in response to a hyperpolarizing, and a regular firing pattern, to a depolarizing square current pulse. Scale bars: 40 pA and 5.3 and 12 mV for the hyperpolarizing and depolarizing membrane potential, respectively, and 50 ms. **(C)** An averaged EPSP (red) in response to low frequency (0.125 Hz) APs in the presynaptic neuron. Scale bars: 0.2 and 76 mV for the EPSP and AP, respectively and 10 ms. **(D)** Temporal modulation of the EPSP amplitudes during trains of APs at increasing frequencies. APs were marked by the black strokes. Intra-train frequencies were 10, 20, and 40 Hz from top to bottom. **(E)** Summary of the geometric distance of synaptic contacts (open squares) and their average (solid squares) and SD (solid line). The lower panel shows the number of synaptic contacts per connection as determined from 3D Neurolucida (*n* = 8) reconstructions with an average of 3.5 ± 0.5 (SD) contacts. **(F)** Two electron microscopically identified *en passant* synaptic boutons (sb) established on different locations on basal dendrites (de) of the postsynaptic pyramidal cell depicted in **(A)**. The two arrowheads indicate the synaptic apposition zones. Scale bar 0.1 μm.

For the reconstruction of individual L5B synaptic boutons and their postsynaptic target structures, three different experimental approaches were used: (1) immersion-fixed acute slices containing the recorded and biocytin- or HRP-filled excitatory L5B thick-tufted pyramidal neurons which were then further processed according to the protocol described below. This experimental approach enables the reconstruction and further quantification of L5B synaptic boutons on electrophysiologically and morphologically identified synaptically coupled excitatory L5B thick-tufted pyramidal neurons. One major disadvantage, however, is that the conversion of biocytin into an electron dense DAB-reaction product obscures the entire PSD and in most cases also the synaptic vesicle pool, two critical and important structural parameters for synaptic transmission and plasticity. Hence, the biocytin-filled neurons were only used for the light microscopic analysis of the number, location and geometric distance of synaptic contacts. To circumvent this problem single and pairs of L5B thick-tufted pyramidal neurons were filled with HRP that does not obscure the interior of the neurons compared to biocytin (see Figures 1F, 4A). Another critical factor is the time of the electrophysiological experiment and subsequent immersion-fixation that could lead to alterations in the preservation of the ultrastructure. Thus, two additional experimental approaches were used: (2) perfusion-fixed brains of eight adult Wistar rats embedded for conventional EM, without any prior manipulations. This guarantees an optimal ultrastructural preservation, and (3) perfusion-fixed brains of three adult Wistar rats where pre-embedding glutamine synthetase immunohistochemistry was carried out to investigate the astrocytic coverage of synaptic boutons and their postsynaptic target structures (Rollenhagen et al., 2015). To look for intra- and inter-individual differences, not only different animals but also different tissue blocks of the same animal were examined.

For the experiments described under 2 and 3, animals were deeply anesthetized with sodium pentobarbital (Narkodorm™, 60 mg/kg body weight) and then transcardially perfused with physiological saline followed by an ice-cold PB-solution containing 4% paraformaldehyde and 0.1–0.5% glutaraldehyde (Polyscience Europe GmbH, Eppelheim, Germany) for 20–25 min. After 1 h of post-fixation, brains were removed from the skull and stored overnight in fresh fixative at 4°C. Serial 200-μm-thick vibratome sections (VT1000S Leica Microsystems, Nussloch, Germany) were cut in the frontal plane through the “barrel field” of the somatosensory neocortex. After incubation for 1 h in sucrose-PB containing 1% osmium tetroxide, sections were washed in PB, and dehydrated in ascending series of ethanol to absolute ethanol. Sections were transferred to propylene oxide (2x2 min), then to a mixture (1:1, 1:2) of propylene oxide and epoxy resin (Durcupan™; ACM, Fluca, Sigma-Aldrich Inc., USA) for 1 h, and then to pure Durcupan™ overnight. Finally, sections were flat-embedded in Durcupan™ and polymerized at 60°C for 2 days.

#### Glutamine synthetase pre-embedding immunohistochemistry

To examine the topography of the astrocytic coverage at L5B synaptic boutons three adult Wistar rats were transcardially perfused with PB-buffered 4% paraformaldehyde and 0.1% glutaraldehyde. After post-fixation, 100 μm vibratome sections were cut in the frontal plane. The barrel field of the somatosensory cortex was dissected from the sections and then cryoprotected in PB-buffered 10% (30 min), 20% (30 min), and 30% sucrose overnight. Sections were then freeze-thawed in liquid nitrogen, rinsed in PB, blocked in phosphate-buffered saline (PBS) containing 0.5% goat serum albumin (1.5 h) and finally incubated in a monoclonal mouse anti-glutamine synthetase antibody (1:1,000; Chemicon Europe, Hampshire, UK) overnight at 4°C. After several rinses in PBS, sections were incubated in biotinylated anti-mouse secondary antibody for 2 h (1:100, Vector, Linaris, Wertheim, Germany). This was followed by several washing steps in PBS and by incubation in PBS-buffered ABC-elite solution for 2 h (1:100; Vector, Linaris, Wertheim; Germany). Sections were then reacted in 0.05 M Tris-buffered DAB for 10 min. After several rinses in PBS they were post-fixed in sucrose-PBS buffered 0.5% osmium tetroxide (30 min), dehydrated through an ascending series of ethanol, propylene oxide, and finally flat-embedded in Durcupan™. Again care was taken to cut serial sections through the basal dendritic domain of a L5B pyramidal neuron located under an individual barrel.

#### Double glutamine synthetase GABA pre-embedding immunohistochemistry

Adult Wistar rats (*n* = 2) were deeply anesthetized and then transcardially perfused as described above. Brains were post-fixed and then serial 150 μm thick coronal vibratome sections were cut. Prior to immunohistochemistry sections were cryoprotected (see above). Sections were freeze-thawed in isopentane cooled with liquid nitrogen for 45 s and rinsed several times in PBS at room temperature. Afterwards, slices were pre-incubated in blocking solution (PBS containing 0.5% BSA) for 30 min at room temperature.

Double immunohistochemistry was carried out as follows: First, a standard immunoperoxidase method was used for the detection of glutamine synthetase. After pre-incubation sections were transferred into mouse anti-glutamine synthetase (1:2,000) and rabbit anti-GABA (1:2,500) diluted in PBS containing 0.5% BSA for 2 days at 4°C. After thorough washes, sections were incubated in the secondary antibodies; a biotinylated anti-mouse IgG antibody to detect glutamine synthetase (1:200) and a gold-labeled rabbit anti-GABA (Fab' fragment, 1:100, Sigma-Aldrich Chemie GmbH, Steinheim, Germany) diluted in the same buffer at room temperature for 2 h.

To distinguish between both antibodies two different detection systems were used. For the identification of glutamine synthetase sections were processed using the ABC-Elite kit as described above. After several washing steps sections were post-fixed in 1% glutaraldehyde diluted in PB for 10 min at room temperature. This was followed by several washes in double-distilled water. To identify GABA, a silver intensification was used (HQ Silver Kit Inc., Yaphank, NY, USA). Sections were incubated for 8 min in the dark and subsequently washed in PB. Finally, sections were processed for conventional EM as described in detail above.

#### Serial sectioning

Prior to serial ultrathin sectioning, semithin sections were cut, toluidine-blue stained and examined light microscopically to trim a block that contains L5B. Serial ultrathin sections (55 ± 5 nm in thickness, silver to light gray interference contrast appearance) were cut on a Leica UltracutS ultramicrotome (Leica Microsystems, Vienna, Austria) and collected on Formvar-coated slot copper grids. A series comprised 80–200 ultrathin sections to allow the reconstruction of individual basal dendritic segments and terminating synaptic boutons.

In case of the synaptically coupled pairs of L5B thick-tufted pyramidal neurons serial ultrathin sections were cut through the basal dendritic domain of the pre- and postsynaptic neurons (*n* = 2). For comparison with the conventionally embedded material, synaptic boutons contacting dendritic segments of the HRP-labeled neurons and the surrounding neuropil were also fully 3D reconstructed.

#### 3D-volume reconstructions

Basal dendrites of L5B thick-tufted pyramidal neurons in all experiments performed (see above) were photographed from the series of ultrathin sections at a primary magnification of x8000 with a Zeiss Libra 120 (Fa. Zeiss, Oberkochen, Germany) equipped with a Proscan 2K digital camera and the SIS analysis software (Olympus Soft Imaging System, Hamburg, Germany). The digital images were then imported into the reconstruction software OpenCAR (Sätzler et al., 2002), stacked and transformed linearly such that corresponding structures were aligned along all consecutive images comprising the 3D-image stack (for details see Sätzler et al., 2002). In each image series comprising a 3D-stack, all structures of interest were marked using closed contour lines in OpenCAR. From these polygonal cross-sections, 3D-volumetric reconstructions were performed from which surface and volume measurements were obtained. Within a given stack only “complete” synaptic boutons were included in the sample and were selected by the following criteria: endterminal boutons: in most of the cases part of the axons could be followed throughout the series of digital images leading into the opening of a synaptic bouton. The termination was initiated by the decrease in size until the structure became too small to be visible in the following consecutive digital images. *En passant* boutons: here the axon and bouton could be followed in both directions within a series of ultrathin sections.

In addition, the surface areas of the pre- (PreAZ) and postsynaptic density (PSD) were measured. PreAZs and PSDs were marked as 2D contour lines in each cross section and their surface areas (SA) were computed separately using the following steps. First a triangulated 3D surface model of the synaptic bouton was generated and its surface area measured by summing the areas of all triangles contributing to the model. A PreAZ surface model was then generated by extracting those triangles from the 3D surface model of the synaptic bouton that were located close (i.e., within a 30 nm distance) from its 2D contour line. Hence the length (L) of the PreAZ (L PreAZ) and the surface area of the PreAZ (SA PreAZ) is already known. Finally, the size of the PSD opposing the PreAZ was estimated under the following assumptions: (1) both membrane specializations, PreAZ and PSD run parallel to each other at the pre- and postsynaptic apposition zone; (2) for both membrane specializations a contour line was drawn determining their actual length (L PreAZ and L PSD). Hence the surface area of the PSD (SA PSD) is estimated by the following equation:

$$\begin{array}{l} \text{SAPSD}=\text{SAPr}{\text{e}}^{*}LPSD/LPreAZ \end{array}$$

that is the perimeter ratio between the outlines of the PSD to that of the synaptic contact.

The synaptic cleft width was measured at the two lateral edges and the center of the PreAZ and PSD on digital EM images using the SIS analysis software. Only synaptic boutons in which the AZ was perpendicularly cut and which showed the typical broadening of the synaptic cleft were included in the sample (*n* = 5 animals, *n* = 155 AZs). The two values for estimating the cleft width for the two lateral edges were averaged and a mean ± SD was calculated for each animal. Finally, a total mean ± SD over all animals was given.

#### Analyzing vesicle distribution and pool sizes

To estimate the number and size of clear synaptic and dense-core vesicles (DCVs), all vesicles were marked throughout each synaptic bouton and their diameters were individually measured. To determine the distribution of vesicles, the minimal distance between each vesicle membrane and the projection of the contour lines of the PreAZ on the boutons membrane was measured in two dimensions. To avoid double counts, DCVs were measured only in the section where they appear largest. All calculations were performed off-line using a batch version of OpenCAR. For additional information on 3D-reconstruction, see also Sätzler et al. (2002) and Rollenhagen et al. (2015).

#### Tissue fixation and preparation

In this study aldehyde fixation was used that is thought to induce tissue shrinkage thereby biasing structural quantification (but see Eyre et al., 2007; Korogod et al., 2015). A direct comparison of structural parameters obtained from either aldehyde or cryo-fixed and substituted tissue samples (Korogod et al., 2015), had shown differences in extracellular and glial volume, but no significant differences in surface area and volume of axons, dendrites, synaptic boutons, and other synaptic subelements such as mitochondria, AZs and synaptic vesicles were observed (see also Zhao et al., 2012a,b). Therefore no corrections for shrinkage were applied and we are thus convinced that the synaptic parameters reported here are accurate and can be directly used in detailed computational models.

#### Cluster analysis

Hierachical cluster analysis (HCA) was performed using R (v.3.2.2, The R Foundation for Statistical Computing, https://www.r-project.org). All synapses reconstructed in this study (*n* = 148) were clustered based on multiple structural parameters, namely bouton surface area and volume, number of AZs/bouton, number of mitochondria, volume of mitochondria, percentage of mitochondria volume, number of vesicles, total vesicular volume, percentage of vesicular volume, vesicle diameter, number of vesicles at distances 10 / 20 / >20 – <60 / 60 / 100 / 200 / 60-200 / >200 nm from the PreAZ, AZ surface area, PSD surface area and a categorical value determining their location on a spine or dendritic shaft. Care was taken to select non-overlapping parameters for each analysis; thus we selected vesicle numbers at non-overlapping distances from the PreAZ, either absolute or percentage mitochondria volume and either absolute or percentage vesicles volume (see also Supplementary Table 1).

HCA is a multivariate technique to arrange data points that are characterized by a large number of variables (in the present case, structural parameters of individual synaptic boutons) into agglomerative clusters. Analysis parameters and cluster methods need to be selected that prescribe how the high-dimensional (dis-)similarity or distance of data points is calculated, and how data are joined into clusters. For the present analysis, we used the general approach of Ward's method (Ward, 1963), which, at each step of merging clusters, attempts to minimize the total within-cluster variance, based on the squared Euclidean distance between cluster centers. The results were corroborated by further multivariate analyses described below.

#### Multidimensional scaling

MDS is a multivariate technique that takes a set of high-dimensional dissimilarities (i.e., distances) and transforms them into a configuration of points in a lower-dimensional representation (typically, 2D or 3D), such that the distances between the points are approximately equal to the high-dimensional dissimilarities. In this way, the technique allows exploration and visualization of similarity relations among data points, such as communities or gradients.

#### Density plots

We used density plots to graph the distribution of the morphological parameters for the two principal classes of synapses. The approach estimated the kernel densities based on Gaussian kernels.

#### Box plots

To look for inter-individual differences between animals for each structural parameter, data distributions were analyzed using boxplots (see Supplementary Figures 2–4).

#### Non-parametric comparisons

We used the non-parametric Kruskal-Wallis rank sum test to compare the morphological parameters of synaptic boutons belonging to the two main classes that were identified by HCA. Differences were assessed by the Chi-squared test parameter, and significance was evaluated after Bonferroni correction for multiple comparisons. We pre-assigned a two-tailed significance level of *p* = 0.05 (uncorrected) to all tests.

#### Statistical analysis

From the numerous 3D-reconstructions and spreadsheets computed by OpenCAR, statistical summaries and graphs were generated automatically using special purpose functions written for the statistics package. All calculations were performed offline using a batch version of OpenCAR, which generates 3D-reconstructions as well as space-delimited tables for each measurement that are readable by standard analysis software. For further details on 3D-reconstructions see Sätzler et al. (2002).

To assess differences in the distributions for the size of the PreAZs and PSDs, the non-parametric Kolmogorov-Smirnov test was used. In addition, the non-parametric Kruskal-Wallis H-test with Dunn's *post-hoc* analysis was used to look for differences between animals for the structural parameters analyzed. Results were considered significant if *p* < 0.05. Where appropriate, p values were explicitly specified. For all values mean ± SD over all animals and the median with the interquartile range is given. Moreover, the skew and kurtosis is indicated as some of the parameters investigated have shown a non-normal distribution (see Tables 2, 3). For all correlations the Spearman correlation factor (r_s_) is given.

For publication selected EM images were further processed using Adobe Photoshop™ and Adobe Illustrator™ software packages.

## Results

### Variable synaptic transmission between pairs of excitatory L5B thick-tufted pyramidal neurons

Multi whole-cell patch-clamp recordings were made from synaptically coupled L5B thick-tufted pyramidal neurons (2–4 simultaneously recorded neurons, *n* = 25 connections from 17 rats) in the somatosensory cortex of young adult rats (Figure 1A). L5B pyramidal neurons are distinguishable from slender tufted L5A pyramidal neurons by the different location, shape, and size of their somata, main apical trunk, apical oblique, and basal dendrites and electrophysiological properties (Markram et al., 1997a,b; reviewed by Ramaswamy and Markram, 2015). L5B thick-tufted pyramidal cells exhibited a characteristic sagging membrane potential during strongly hyperpolarizing stimuli and a regular spiking pattern in response to suprathreshold currents (Figure 1B). Single action potentials (APs) and high-frequency trains were evoked in the presynaptic neurons to study the stationary and temporally modulated excitatory postsynaptic responses (EPSPs), respectively (Figures 1C,D). During recordings, the pre- and postsynaptic neurons were filled with biocytin or HRP to reveal their morphology and to analyze the density, distribution and geometric location of their synaptic contacts (Figures 1A,E,F). Synaptically coupled HRP-filled pairs and single HRP-filled L5B pyramidal neurons were further investigated at the EM level to 3D reconstruct and quantify synaptic boutons terminating onto functionally and morphologically identified excitatory L5B thick-tufted pyramidal neurons (Figures 4, 5).

An average of 3.5 ± 0.5 synaptic contacts per connection was distributed over the entire basal dendritic tree at a mean geometric distance of 69.6 ± 20.2 μm (28 contacts from 8 connections; min: 28.9 μm; Max: 103.6 μm) from the soma (Figure 1E). The majority of synaptic contacts were established on 3rd (53.6%), the remaining on 4th (25.0%) and 2nd (21.4%) order basal dendrites. In addition, some of the HRP-labeled presynaptic boutons and postsynaptic dendrites were verified by high-resolution fine-scale EM (Figure 1F).

### *P_r_* and quantal analysis at excitatory L5B-L5B synaptic connections

**Figure 2:**
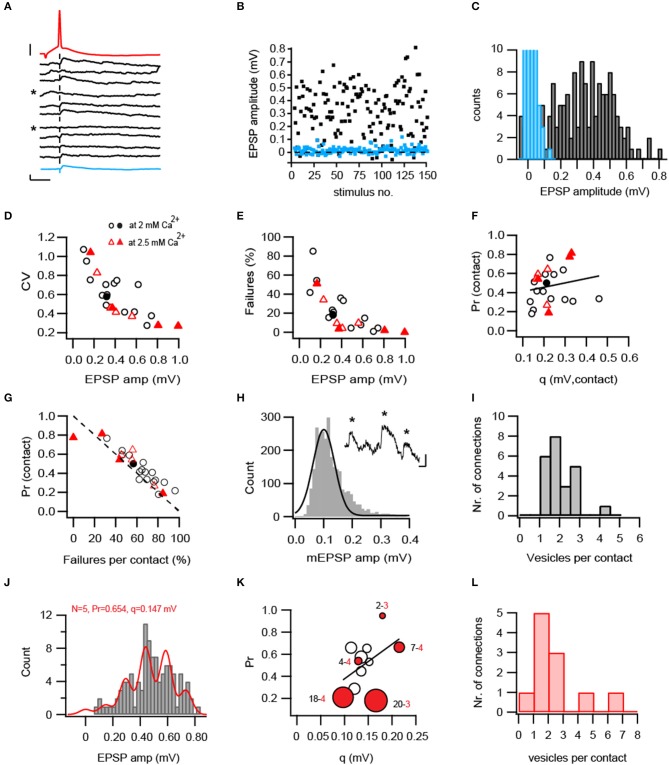
Functional properties of L5B-L5B pyramidal cell connections. **(A)** Consecutive EPSPs (black) in response to presynaptic APs (red) evoked at 0.125 Hz. The average EPSP of 30 trials is shown below as a thick blue trace. Failures to evoke an EPSP were denoted by asterisks. Scale bars: 1 and 20 mV for the EPSP and AP, respectively and 10 ms for both. **(B)** EPSPs (black squares) were stationary over 148 trials but fluctuated strongly in amplitude and failures (blue squares) were often observed (18%). **(C)** Broad distribution of EPSP amplitudes (black bars) showing the absence of clearly separated peaks in their histogram. Blue bars represent background noise, curtailed for better visibility. **(D–E)** CV of the EPSP amplitudes **(D)** and percentage failures **(E)** decrease in connections with larger EPSP amplitudes (*n* = 25). Filled symbols represent connections for which the number of synaptic contacts was determined from the 3D-reconstructions. **(F)** Absence of correlation between *P*_*r*_ and q at individual synaptic contacts (*R* = 0.19, *R*^2^ = 0.036, *P* = 0.39). Note that contacts with large q have either a low or high *P*_*r*_. **(G)** *P*_*r*_ (calculated from the CV) is strongly anti-correlated with the independently-measured percentage failures per contact (*R* = −0.824, *R*^2^ = 0.67, *P* < 10^−5^). The unity line is shown in a dashed stroke. **(H)** The vesicular quantal size estimated from measurements of spontaneous fast mEPSPs impinging onto L5B neurons (examples in inset are denoted by asterisks). A Gaussian function was fitted with a median of 0.113 mV and SD = 0.053 (*n* = 2803 events from 7 neurons). **(I)** Estimates of synchronous vesicular release. Between 1 to 4 vesicles are released from individual contacts during a single AP. **(J–L)** Binomial quantal parameters obtained from model fitting to the EPSP amplitude histogram. **(J)** Example of a successful fit (red line) depicting distinctive peaks in the EPSP amplitude histogram. **(K)** Model estimates from 11 successfully-fitted connections*. P*_*r*_ and q are displayed in the Y- and X-axis, respectively. Estimated N is displayed as a function of marker size (small-to-large). Red-filled circles mark the anatomically reconstructed connections. The numbers next to them denote the model-estimated number of release sites (black) and anatomically determined number of contacts (red). Linear correlation between *P*_*r*_ and q was non-significant (*R*^2^ = 0.209, *P* = 16). **(L)** Estimates of number of vesicles released per contact based on the quantal binomial fits. The model-estimated N was divided by the averaged (4) or connection-specific number of anatomically identified contacts.

To investigate the stochastic release process at L5B-L5B excitatory synaptic connections, series of presynaptic APs (30–180) were elicited at low-frequency (0.125 Hz) and the resulting EPSP amplitudes were measured. Within individual connections EPSPs exhibited significant fluctuations in amplitudes and occasional failures (see example in Figures 2A–C and Table 1) that were reflected in relatively high coefficient of variations (CVs) and percentage failures (F%; see also Figures 2D,E). Across all connections, CVs and failures varied several folds and were negatively correlated with the mean EPSP amplitude, indicating that weak connections were highly variable while strong ones were more reliable (Figures 2D,E and Table 1). The quantal binomial model of release is often used to infer the number of available transmitter quanta (N), their *P*_*r*_ and quantal size (q) from fluctuation analysis of the EPSP amplitude. To estimate these parameters at a single synaptic contact, we have undertaken two approaches. First, we performed a simple calculation of *P*_*r*_ and q from the CVs, based on the quantal binomial equations assuming that the number of synaptic contacts per connection was 4 (the actual number determined from the 3D Neurolucida reconstructions, Figure 1A) and that all contacts had the same *P*_*r*_ and q. The calculated *P*_*r*_ and q varied roughly 4-fold between individual connections and were each positively correlated with the EPSP amplitude (Supplementary Figures 1A,B) suggesting that both *P*_*r*_ and q contribute to strengthening synaptic transmission. However, at individual synaptic contacts *P*_*r*_ and q were not significantly correlated with each other (Figure 2F, *R*^2^ = 0.036, *P* = 0.39), suggesting that they are independently controlled. Accordingly, at higher extracellular calcium *P*_*r*_ was increased and CVs and F% were reduced, but q remained unchanged (Table 1).

**Table 1:** Physiological parameters of adult L5B-L5B excitatory synaptic connections.

		**EPSP amp (mV)**	**CV**	**F%**	***P_r_*^#^**	**Q^#^ (mv)**
at 2 mM [Ca^2+^]_o_	Mean ± SD	0.39 ± 0.20	0.62 ± 0.20	25.21 ± 21.00	0.43 ± 0.16	0.23 ± 0.09
	Range (min-max)	0.1–0.74	0.28–1.07	1–81	0.18–0.77	0.14–0.46
	n	15	17	17	17	15
at 2.5 mM [Ca^2+^]_o_	Mean ± SD	0.49 ± 0.29	0.52 ± 0.27	14.26 ± 18.34	0.55 ± 0.22	0.23 ± 0.07
	Range (min-max)	0.1–0.74	0.27–1.04	0–51	0.19–0.81	0.16–0.33
	n	8	8	8	8	8

# *Calculated from the CV with the assumption of N = 4 contacts per connection or the anatomically determined N*.

The observed F% were negatively correlated (*r* = −0.828, *R*^2^ = 0.67, *P* < 10^−5^) with *P*_*r*_ and mostly aligned along the unity line (dashed line in Figure 2G) as expected from the binomial quantal model.

In each synaptic contact, q represents the averaged size of the transmitter quanta released per AP. Yet, to how many synaptic vesicles does this quantal size correspond? To answer this question we measured spontaneous fast-rising miniature EPSPs (mEPSPs) that likely represent q, the release of a single synaptic vesicle. The mEPSP amplitude was 0.113 ± 0.005 mV (Figure 2H, median ± SD, 2803 events from 7 neurons) and was termed q_vesicle_. The q calculated per contact was divided by q_vesicle_ suggesting that 1–4 synaptic vesicles were released from individual synaptic boutons during single APs (Figure 2I). Note that this conclusion deviates from the original single-vesicle assumption of the binomial release model and could only be congruent with it if all vesicles are released simultaneously and *P*_*r*_ is determined for the entire AZ rather than for individual vesicles.

An alternative approach to estimate quantal parameters without making prior assumptions on the number of release sites or q, is to fit EPSP amplitude histograms with multiple Gaussians. We fitted EPSP amplitude histograms with the binomial model using a previously published algorithm (Hardingham et al., 2006, 2010; developed and made available by Jenny Read at www.jennyreadresearch.com). An example of a successful fit is shown in Figure 2J, depicting regularly distributed peaks corresponding to 5 release sites and quantal amplitude of 0.147 mV and *P*_*r*_ of 0.654. Although amplitude peaks were not always clearly separated, for 11 out of 25 connections a satisfactory fit to this model was obtained when AP conductance failures were incorporated in the model in addition to quantal release failures (Supplementary Figure 1). As in the first approach, estimated *P*_*r*_ varied several folds from 0.18 −0.95 (Figure 2K) with an average of 0.50 ± 0.23 (Supplementary Figure 1G). However, q displayed a narrow distribution with a median of 0.135 mV and a range 0.097–0.215 mV (Figure 2K) that corresponds to 1 to 2-folds the value of q_vesicle_. The correlation between *P*_*r*_ and q was weak and non-significant (*R*^2^ = 0.209, *P* = 0.16). N ranged from 1 to 20 and was on average twice the number of anatomically-determined contacts (Model average 8.4 ± 6, anatomical average: 3.5 ± 0.5); or much higher in some connections (red circles in Figure 2K and Supplementary Figures 1D,F). The likeliness of finding as many as 20 contacts per connection in the 3D-reconstructions is very low; thus, we conclude that the algorithm estimated parameter N represents the number of vesicles in the entire connection rather than the number of synaptic contacts. Dividing N by the number of anatomically identified contacts (Figure 2L) yielded an average of 2.28 vesicles per contact (range 0.7–6.7), a number similar to that obtained with the first approach. We note that quantal parameters obtained with this method should be considered with caution given the high estimates of N and *P*_*r*_, compared to the anatomically-measured contacts and independently detected failure percentage.

Taken together, quantal estimates with two analysis methods indicated multivesicular release from individual L5B-L5B synaptic boutons and uncorrelated *P*_*r*_ and q.

### Temporal dynamics of synaptic transmission and functional vesicle pools

**Figure 3:**
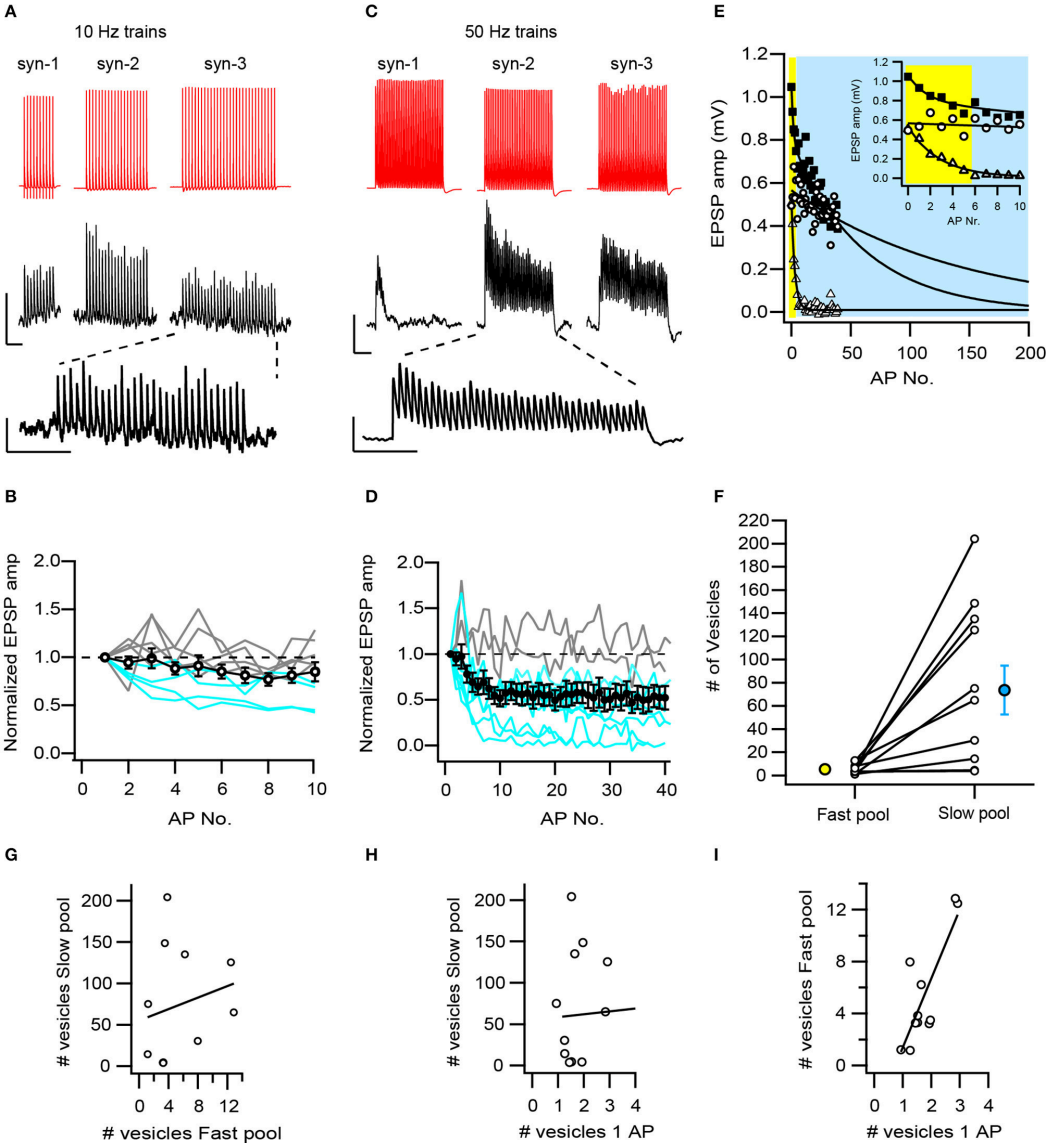
Temporal dynamics of L5B-L5B synapses are generated by different combinations of vesicle pools. **(A)** Constant synaptic responses are commonly observed during low-frequency trains (10 Hz) of APs. Averaged EPSP trains from three synaptic connections show constant or slightly depressing amplitudes. A typical EPSP train is shown enlarged. Scale bars: 47 and 0.4 mV for the AP and EPSP, respectively and 500 ms, same for the enlarged EPSP train. **(B)** L5B-L5B synapses typically exhibit constant synaptic transmission during 10 Hz trains of APs. Open circles represent the averaged responses of 11 synaptic connections, individual facilitating and constant connections are represented by gray curves and depressing connections by the blue curves. **(C)** Depressing synaptic responses dominate at high-frequency (50 Hz) trains. Averaged EPSP traces from 3 synaptic connections (same as **A**) exemplify different response patterns: rapidly, slowly- and non-depressing EPSP amplitudes. A typical pattern is plotted enlarged. Scale bars: 35 and 0.4 mV for the AP and EPSP, respectively and 200 ms, same for the enlarged EPSP train. **(D)** On average, EPSPs depressed mildly during high-frequency trains (black circles with error bars, *n* = 11). However, the individual synaptic responses vary from facilitation (gray curves) to strong depression (blue curves). **(E)** Fit of the EPSP amplitudes during high-frequency trains to a double-exponentially decaying function with a fast and a distinct slow time constants. Three individual synapses are plotted and fitted (triangles, squares, and circles for syn-1, syn-2, and syn-3, respectively). The fitted area under the fast and slow exponential decay time constants are shaded yellow and blue, respectively. The inset shows the first 10 EPSPs in the train. Note, that in each synapse the fitted area was dominated either by the fast or the slow processes or by both. **(F)** Two pools of vesicles account for the temporal dynamics of L5B-L5B synapses during high-frequency trains: a rapidly recruited and depleted pool containing on average 5.4 (yellow circle) and a slowly depleted pool containing 74 (blue circle) vesicles. At individual synapses (open circles) the slow pool varied dramatically in size. **(G)** The fast and slow vesicular pools are not correlated with each other at individual synapses (*R*^2^ = 0.04, *P* = 0.55). **(H)** The slow pool is not correlated with the number of vesicles released during a single AP. **(I)** The number of vesicles released during a single AP is highly correlated with the size of fast pool (*R*^2^ = 0.67, *P* = 0.002).

During sensory stimulation and execution of complex behaviors as well as Up-states (Zhou and Fuster, 1996; Sanchez-Vives and McCormick, 2000; Sakata and Harris, 2009), cortical L5B thick-tufted pyramidal neurons fire multiple APs at high frequencies. During such prolonged and intense activity, synaptic transmission could be modulated in various ways depending on the availability of synaptic vesicles and on their recycling rates. To investigate these temporal modulations and their underlying mechanisms, long trains of presynaptic APs were repeatedly evoked at either 10 or 50 Hz intra-train frequencies and the EPSPs were measured. In response to 10 Hz stimulation, most connections exhibited constant EPSP amplitudes even after 10 APs (5/9 connections, Figures 3A,B) or 30 APs (Figure 3A syn-3). In 2 cases a mild and in 2 others a stronger depression of the EPSP amplitudes was observed. The averaged curve of EPSP amplitudes shows that L5B-L5B synapses are easily capable of supporting prolonged release at 10 Hz. In contrast, increasing the intra-train frequency to 50 Hz, resulted in an accumulating depression of the EPSP amplitudes in most connections (9/11 connections). Depression started within the first 3 APs and developed to a steady state level by the 10th AP. The steady state degree of depression varied from strong to mild and in 2 cases EPSP amplitudes remained constant even after 40 APs at 50 Hz (Figures 3C,D). These results show that at high firing frequencies and ongoing release, synaptic resources often become limited yet to a variable extent.

A major reason underlying accumulating depression is the depletion of the RRP of synaptic vesicles at a rate faster than its replenishment. To estimate the size of the RRP we have adapted the method of Dobrunz and Stevens (1997) and fitted the EPSP amplitudes of the 50 Hz trains with a zero-approaching exponential function (Figure 3E). All synapses were fitted by a sum of two exponentials, a fast and a slow one. Typically, both terms contributed substantially to the decay (Figure 3E filled squares), in some, the slow term dominated (Figure 3E open circles) while in others the fast one (Figure 3E open triangles). The fast and slow decay constants differed strongly (3.6 ± 0.8, range 1.2–8.8 APs and 155 ± 47 APs, range 29–418 APs, respectively, *n* = 10) suggesting that they describe two distinct processes, for example, the depletion of two different pools of vesicles. To estimate the number of vesicles in these two presumed pools, we calculated the area under the exponential fit and divided it by the number of contacts per connection and by the quantal vesicular size (0.113 mV, see Figure 2J). The results suggest that the “fast pool” and the “slow pool” consist of 5.40 ± 1.24 synaptic vesicles (*N* = 11, range 1.2–12.8, Figure 3F) and 74 ± 21 (*N* = 11, range 3.4–204, Figure 3F), respectively. Although the “slow pool” is larger than the “fast” one by ~12-fold (*P* = 0.008, paired *T*-test) no clear linear correlation exists between them across all synapses (Figure 3G, *R*^2^ = 0.04, *P* = 0.55). Likewise, no correlation was observed between the “slow pool” and the number of vesicles released during a single AP (Figure 3H, *R*^2^ = 0.06, *P* = 0.48). In contrast, the latter was strongly and positively correlated with the “fast pool” (Figure 3I, *R*^2^ = 0.67, *P* = 0.002).

We hypothesize that the “fast pool” reflects the RRP of vesicles from which docked and primed vesicles are released most promptly during an AP. The “slow pool” might represent the RP of vesicles that can very rapidly (within 100 ms or less, see Figure 3A) replenish the RRP, but whose size is determined independently. Various combinations of these pools exist in the population of L5B-L5B synapses and likely underlie their heterogeneous responses to trains of APs.

In summary, our functional analysis of unitary and temporally modulated EPSPs in adult L5B-L5B excitatory synapses shows a high heterogeneity of EPSP amplitudes, CVs, F%, *P*_*r*_, and q, and levels and rates of depletion. We hypothesize that heterogeneous and independent pools of docked, readily releasable, and recycling vesicles underlie these properties and combine to generate the wide range of observed synaptic responses.

We next asked whether the structural organization of synaptic boutons in L5B could explain this functional properties and whether structural correlates could be found to the presumed functional pools of synaptic vesicles.

### Synaptic innervation of basal dendrites of L5B thick-tufted pyramidal neurons

**Figure 4:**
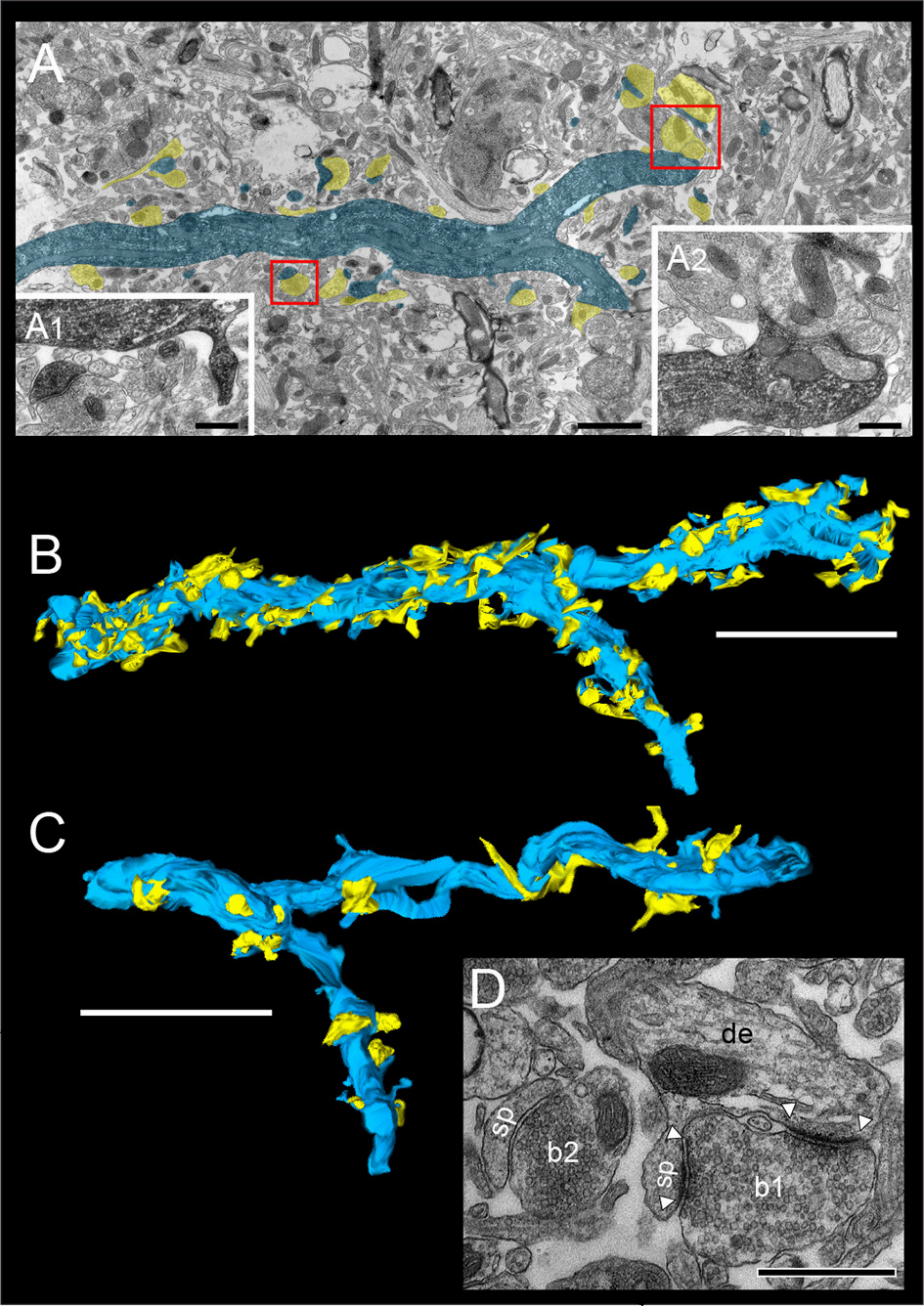
Distribution of L5B synaptic boutons on HRP-filled dendritic segments. **(A)** Low power electron micrograph showing the distribution of synaptic boutons (transparent yellow) on an HRP-filled dendritic segment (transparent blue) of the postsynaptic neuron shown in Figure 1A. Note the high density of synaptic inputs terminating on this dendritic segment. Scale bar: 5 μm. (A1) Synaptic boutons terminating on HRP-labeled spines (framed area in A on the left), (A2) Two shaft synaptic boutons at the HRP-labeled dendrite (framed area in A on the right). Scale bars in (A1,A2) 0.25 μm. **(B)** 3D-volume reconstruction of the dendritic segment shown in **(A)**. The dendrite is given in blue, terminating synaptic boutons in yellow. Note the different shape, size and dense distribution of synaptic boutons (*n* = 199) Scale bar: 2.5 μm. **(C)** Another basal dendritic segment (blue) with a much lower number of synaptic boutons (yellow). Scale bar: 2 μm. **(D)** High power electron micrograph of a synaptic bouton (b1) on both the spine head (sp) and dendritic shaft (de) and an adjacent bouton (b2) terminating on a different spine (sp). AZs are marked by arrowheads. The image was taken in the surrounding neuropil of the HRP-filled pair of neurons. Scale bar: 0.5 μm.

**Figure 5:**
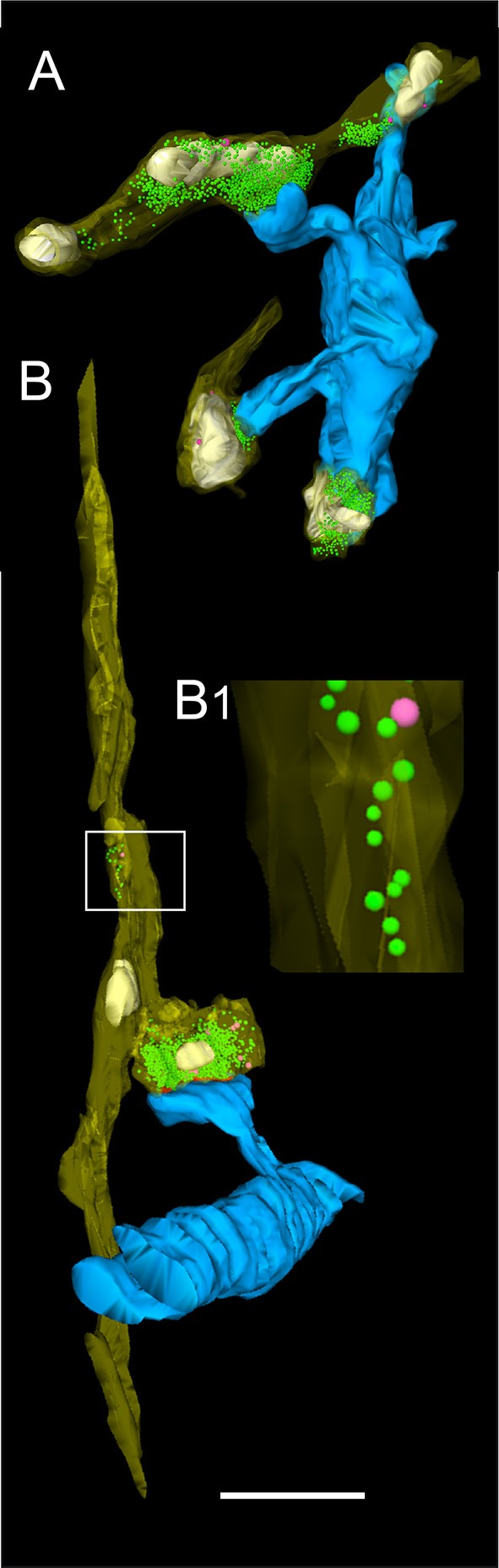
3D-volume reconstruction of synaptic boutons terminating on basal dendrites of L5B thick-tufted pyramidal neurons. **(A)** Quantitative 3D-reconstruction of four synaptic boutons (transparent gold) terminating on three different dendritic spines and a shaft of the same basal dendritic segment (blue). The outline of the bouton was made transparent to visualize the distribution of mitochondria (white), the total pools of synaptic vesicles (green dots) and that of dense-core vesicles (magenta). **(B)** *En passant* axon (transparent gold) that could be followed over long-distance in consecutive electron micrographs establishing an axo-spinous synaptic contact on a postsynaptic dendritic segment (blue). Note the association of mitochondria (white) with the pool of synaptic vesicles (green dots). The framed area shows the presence of synaptic and a dense-core vesicle within the *en passant* axon at higher magnification (B1). Scale bar in **(A,B)** 1 μm.

In this study synaptic contacts were exclusively located on basal dendrites (Figure 1A, but see also Markram et al., 1997a,b). Thus, only basal dendritic segments of functionally and/or morphologically identified L5B pyramidal neurons were completely 3D-reconstructed and quantified (*n* = 30 dendritic segments of various length, *n* = 148 synaptic boutons, see Table 2). In the conventionally embedded material L5B was identified in toluidine-blue semithin sections by the large somata of thick-tufted L5B pyramidal neurons. Basal dendritic segments were selected upon the following criteria: location around and underneath large somata. The apical trunk and oblique trunk dendrites were excluded by their size, location and projection in L5B. Most L5B thick-tufted pyramidal neurons receive dense synaptic input along their basal dendritic tree (Figures 4A,B); the majority (~80%) was established by *en passant* boutons (Figures 4A,B, 5) mainly on dendritic spines of different type (Figures 4, 5). The remaining synaptic boutons terminated on dendritic shafts (Figures 4A,D, 5A). Stubby, mushroom, thin and filopodial-like spines were targeted by one, occasionally by two synaptic boutons (Figure 4D). At ~80% of spines a specialized form of the endoplasmic reticulum, a spine apparatus was found (Figure 11B, see Discussion).

**Table 2:** Quantitative analysis of structural elements of cortical L5B excitatory synaptic boutons.

	**Synaptic boutons**			**Mitochondria**		**Active zones**				
							**Presynaptic**	**Postsynaptic**		
**Animal identity**	**Number of synaptic boutons investigated**	**Surface (μm^2^) ± SD**	**Volume (μm^3^) ± SD**	**Volume (μm^3^) ± SD**	**% of volume**	**Number ± SD**	**Surface area (μm^2^) ± SD**	**Surface area (μm^2^) ± SD**	Cleft width (nm) ± SD^*^ lateral central	
PC020300A p28_I	12	10.65 ± 6.36	0.50 ± 0.47	0.10 ± 0.13	17.13	–	–	–	^*^15.79 ± 1.28	29.87 ± 4.17
PC020300A p28_II	24	6.00 ± 4.99	0.23 ± 0.28	0.05 ± 0.07	11.77	–	–	–	–	–
R-ad 151106_I	12	7.31 ± 3.34	0.29 ± 0.10	0.04 ± 0.02	14.36	1.08 ± 0.67	0.18 ± 0.12	0.16 ± 0.09	15.43 ± 1.32	34.03 ± 4.48
R-ad 170407_I	21	11.31 ± 10.38	0.52 ± 0.42	0.05 ± 0.04	10.47	1.24 ± 0.44	0.16 ± 0.11	0.21 ± 0.13	–	–
R-ad 170407_Id	41	6.83 ± 5.11	0.37 ± 0.26	0.07 ± 0.06	16.38	1.12 ± 0.40	0.27 ± 0.14	0.30 ± 0.15	–	–
R-ad 170407_II	5	4.34 ± 3.91	0.16 ± 0.16	0.03 ± 0.03	11.23	1.20 ± 0.45	0.19 ± 0.10	0.20 ± 0.08	16.69 ± 1.85	31.67 ± 3.22
GS_R_ad 020205_I	5	11.58 ± 3.44	0.92 ± 0.27	0.18 ± 0.13	19.06	1.00 ± 0.00	0.70 ± 0.27	0.76 ± 0.31	15.42 ± 2.16	33.48 ± 3.16
GS_R_ad 070605_I	17	9.91 ± 6.57	0.32 ± 0.22	0.08 ± 0.05	17.53	1.18 ± 0.39	0.33 ± 0.19	0.32 ± 0.19	14.90 ± 1.50	29.98 ± 1.77
GS_R_ad 150805_I	11	4.02 ± 1.46	0.15 ± 0.08	0.03 ± 0.01	17.84	1.00 ± 0.00	0.19 ± 0.12	0.22 ± 0.12	14.88 ± 1.09	28.90 ± 1.51
Mean ± SD	–	8.19 ± 2.84	0.38 ± 0.23	0.07 ± 0.05	15.09 ± 3.22	1.12 ± 0.09	0.29 ± 0.19	0.31 ± 0.21	15.52 ± 0.39	31.32 ± 1.81
Median; IQR	–	8.61; 5.23	0.32; 0.29	0.05; 0.06	16.38; 6.18	1.12; 0.20	0.16; 0.14	0.17; 0.10	15.03; 0.90	30.19; 2.46
CV	–	0.35	0.61	0.71	0.21	0.08	0.66	0.68	0.02	0.04
Skew	–	−0.29	1.67	1.71	−0.40	−0.19	1.74	1.09	1.12	0.34
Kurtosis	–	−1.58	*3.36*	*3.26*	−1.68	−1.59	*3.60*	2.31	1.34	−1.95

*Summary of structural parameters relevant for synaptic transmission and plasticity that have been extracted from the detailed 3D-reconstructions of cortical L5B synaptic boutons. Values are taken from HRP-filled pyramidal neurons (PC), conventionally embedded electron microscopic material (R-ad) and sections processed for glutamine synthetase (GS_R_ad) subsequently embedded for EM. Means ± SDs are given for individual animals. For the HRP-filled neurons values for the AZs are not calculated due to the masking of the AZ by the dense DAB reaction product. Cleft width measurements were taken at the two lateral edges of the pre- and postsynaptic densities. A mean ± SD was calculated from the two values. In addition cleft width at the central region under the pre- and postsynaptic AZs were measured*.

* *Values were taken from unlabeled synaptic boutons of the surrounding neuropil of the HRP-filled slice. Italic numbers represent measurements with a skew or kurtosis over 3, indicating a non-normal distribution*.

At the dendritic segment (Figure 4B), which is ~15 μm in length and ~1,000 μm^2^ in surface area including dendritic spines, ~200 synaptic boutons were counted representing the maximum density in the dendritic segments investigated, while at the majority of the dendritic segments a ~2 to 4-fold lower density of synaptic boutons were found (Figure 4C). The spacing between individual boutons was on average 1.33 ± 0.18 μm (min: 1.20 μm; max: 1.59 μm). Excitatory synaptic boutons were always distinguished by the prominent PreAZ and PSD and the larger size and shape of synaptic vesicles. Based on this criteria thus the majority of synaptic boutons terminating on basal dendritic segments are glutamatergic. Interestingly, ~50% of boutons terminating directly on dendritic shafts turned out to be non-GABAergic.

**Figure 6:**
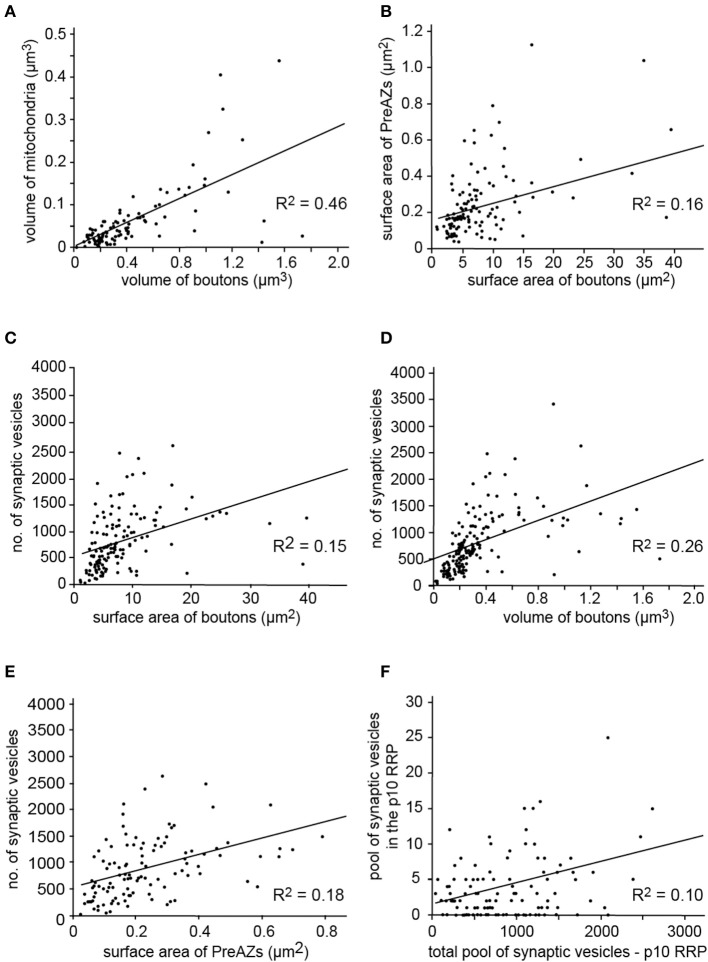
Correlation of several structural parameters of L5B excitatory synaptic boutons. Correlation between the **(A)** Bouton volume and mitochondria showing a good correlation. **(B)** Surface area of boutons with PreAZs which are only weakly correlated. **(C–E)** Surface area of boutons **(C)** the volume of boutons **(D)** and surface area of PreAZs **(E)** showing only a weak correlation with the total number of synaptic vesicles. **(F)** The RRP using the p10 perimeter is not correlated to the total pool of synaptic vesicles (see also Supplementary Figure 5). All data points are fitted by linear regression.

Synaptic boutons were highly variable in shape and size ranging from 0.92 to 39.72 μm^2^ in surface area (average 7.99 ± 2.95 μm^2^) and from 0.02 to 1.73 μm^3^ in volume (average 0.36 ± 0.38 μm^3^, Table 2). All boutons contained either a single mitochondrion, or multiple, relatively large mitochondria that constituted on average 15.09 ± 3.22% to the total volume of the bouton. They were always closely associated with the pool of synaptic vesicles (Figures 5, 8B). A high correlation was found for the volume of boutons with that of mitochondria (Figure 6A; ranged Spearman coefficient of correlation r_s_ = 0.71) suggesting an important role of these structures in the organization and mobilization of the pool of synaptic vesicles at L5B excitatory synaptic boutons.

### AZs at the pre- and postsynaptic apposition zone

The geometry and size of the AZ composed of the PreAZ and the PSD are key structural elements involved in synaptic transmission and in regulating plasticity (Matz et al., 2010; Holderith et al., 2012; reviewed by Südhof, 2012).

The majority (~85%) of synaptic boutons in L5B contained a single, but relatively large AZ. Nearly two thirds of the AZs (~70%) showed perforations in either the PreAZ or PSD (~35%, Figures 8A,C1), the remaining were non-perforated (Figures 8B,C2–C4). Perforated PSDs occurred predominantly on axo-spinous rather than on axo-dendritic synapses (~80%). At a third of spines reconstructed, the AZ were seen to cover nearly the full length of the pre- and postsynaptic apposition zone (Figure 4D).

**Figure 7:**
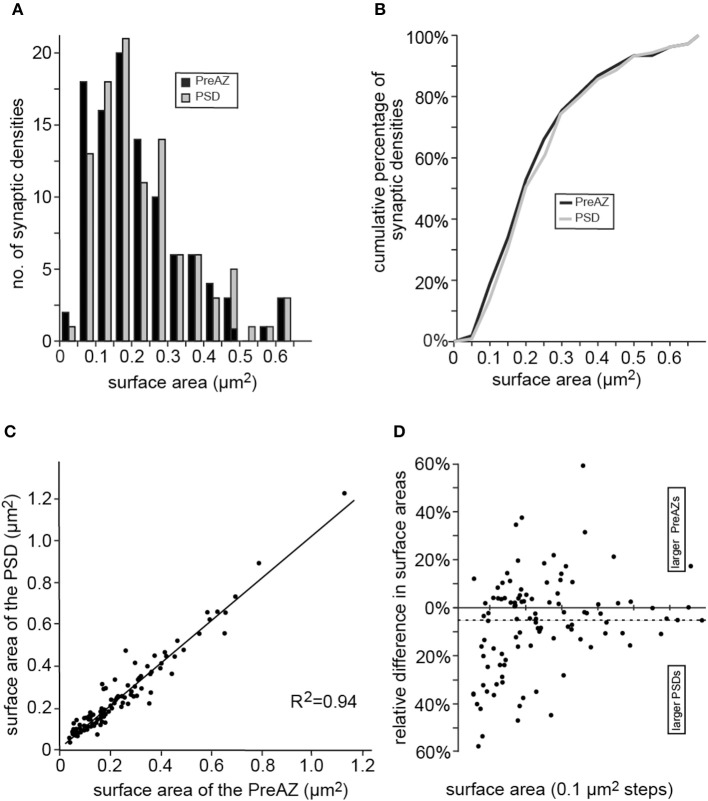
Distribution of the surface areas of PreAZs and PSDs. **(A)** Bar histogram showing the distribution of surface areas for the PreAZ and PSDs. **(B)** Corresponding cumulative histogram. Bin width is 0.01 μm^2^. **(C)** Correlation histogram between the surface areas of PreAZs and PSDs. Data points are fitted by linear regression. **(D)** Dot plot showing differences in surface area between PreAZs and PSDs with a skew to larger PSDs. The dashed line indicates the mean.

Both, the PreAZs and PSDs were highly variable in size with an average of 0.29 ± 0.19 μm^2^ and 0.31 ± 0.21 μm^2^, respectively as indicated by the SD and CV. Beside very large (1.13 μm^2^) also quite small (0.04 μm^2^) PreAZs and PSDs (1.23 μm^2^, 0.04 μm^2^) with a skewness to smaller densities were found (Figures 7A,B, Table 2). The majority (~80%) of the PreAZs and PSDs nearly perfectly matched each other in size (Figure 7C), although ~20% of the PSDs were somewhat larger (Figure 7D).

The cleft width measured under the AZs at the two lateral edges was 15.52 ± 0.39 and 31.32 ± 1.84 nm at the central region; both values were significantly different (*P* ≤ 0.01, Table 2). Only a weak correlation was found between the surface area of PreAZs and that of synaptic boutons (Figure 6B) as indicated by the low *R*^2^ and r_s_ (0.39). Thus, the size of the AZ appears to be independent from that of the synaptic bouton.

### Spatial distribution of synaptic vesicles within the L5B excitatory synaptic boutons

Another key determinant for synaptic efficacy, strength, *P*_*r*_ and plasticity is the size and organization of the pool of synaptic vesicles, in particular, that of the RRP and the RP (Schikorski and Stevens, 2001; Denker et al., 2009; Denker and Rizzoli, 2010; Imig et al., 2014; Schikorski, 2014; Watanabe et al., 2014; for review see Rizzoli and Betz, 2004, 2005; Neher, 2015; Chamberland and Tóth, 2016).

**Figure 8:**
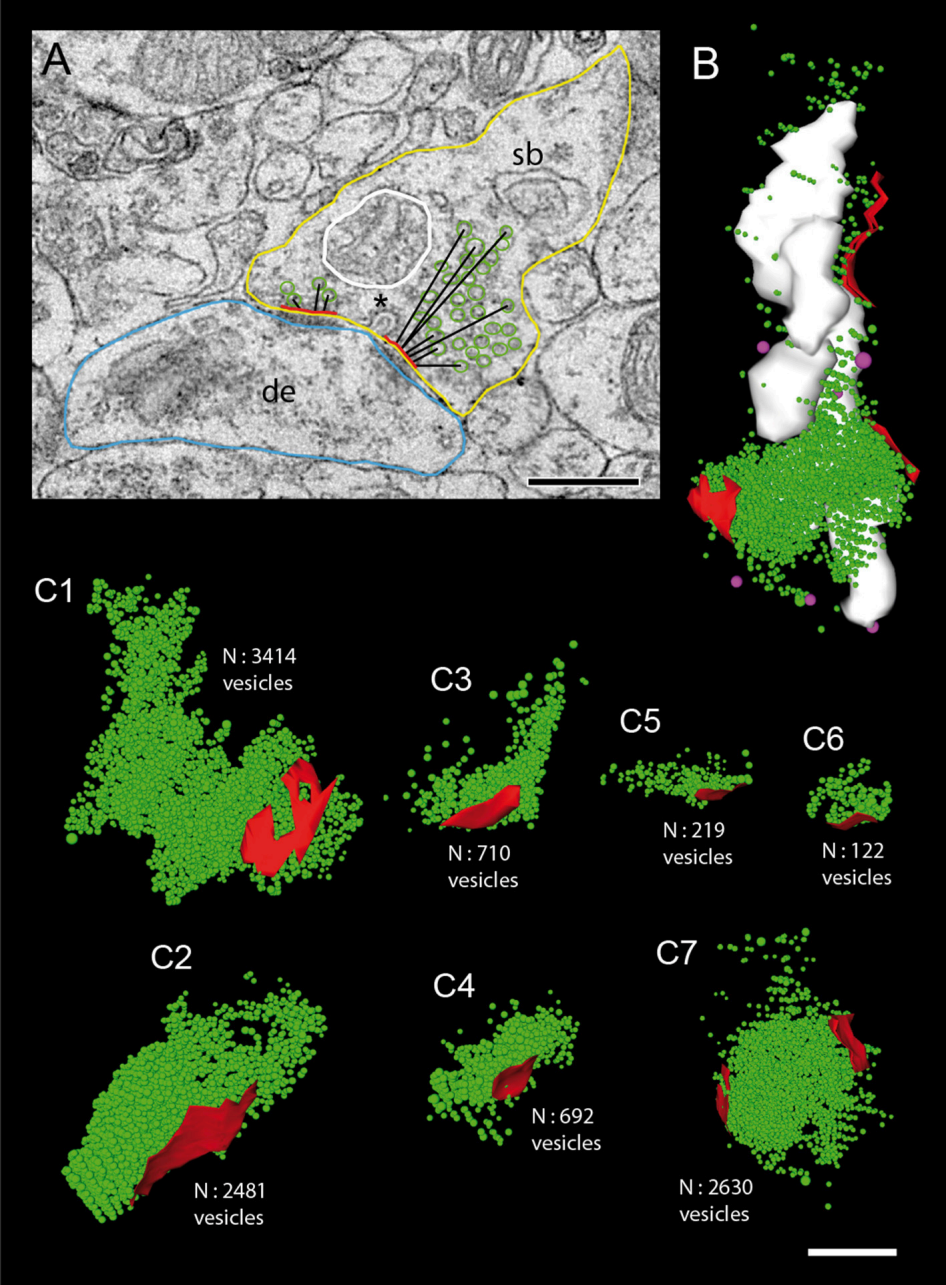
Size of the total pool of synaptic vesicles at individual L5B excitatory synaptic boutons. **(A)** Quantitative analysis of structural parameters relevant for synaptic transmission and plasticity. The following presynaptic parameters were measured: size of the presynaptic bouton (yellow contour), the PreAZs (red lines), and mitochondria (white contour) and the shortest geometric distance from the center of gravity of synaptic vesicles to the PreAZ (black lines) as shown for a subset of vesicles. Note the fused coated pit (asterisk) at the PreAZ. Abbrevations: de, dendrite; sb, synaptic bouton. Scale bar: 0.25 μm. **(B)** 3D-volume reconstruction of the total pool of synaptic (green dots) and dense-core (magenta dots) vesicles distributed at three individual PreAZs (red). Note the arrangement of mitochondria (white) in close proximity to the total pool of synaptic vesicles. **(C1–C7)** 3D-reconstructions of individual total pools of synaptic vesicles (green dots) at PreAZs (red). Note the huge differences in the total pool size between individual synaptic boutons. At one synaptic bouton **(C7)** the pool of synaptic vesicles is shared between two separated PreAZs. No cluster-like arrangements of synaptic vesicles at individual PreAZs were found. Scale bar: 0.25 μm.

In general, synaptic vesicles were distributed throughout the entire synaptic bouton (Figures 4D, 5, 8) and occupied on average 5.78% (mean 0.02 ± 0.02 μm^3^) of the total volume of boutons. Several synaptic vesicles (3–6) were found closely attached or fused (Figure 4D) with the presynaptic membrane pointing to multivesicular release at these synapses.

Two different types of vesicles were found: (1) Clear synaptic vesicles with a mean diameter of 33.75 ± 4.55 nm, and (2) large DCVs (65.19 ± 11.96 nm), which were intermingled with the population of clear vesicles (Figure 8B). Interestingly, some DCVs were seen to fuse with the PreAZ suggesting a role in its build-up (Figure 8A; Shapira et al., 2003; Schoch and Gundelfinger, 2006), and maintain the clustering of synaptic vesicle without directly participating in vesicle exocytosis (Mukherjee et al., 2010).

The total pool of synaptic vesicles was on average 811.47 ± 272.25 ranging from ~50 to ~2,000 in individual boutons (see also Table 3) representing ~6% of the total volume. In our sample boutons with only a small number of synaptic vesicles (Figures 8C5,C6), others with a moderate, but variable number (Figures 8C3, C4) and boutons with a relatively large number of synaptic vesicles (Figures 8C1,C2,C7) were found. Occasionally the pool of synaptic vesicles was shared by up to three PreAZs (Figure 8B).

**Table 3:** Synaptic vesicle pools at cortical L5B excitatory synaptic boutons.

**Synaptic vesicles**						**Pool sizes**									
				**Total volume**		**Presumed RRP** ≤ **60 nm**					**Presumed RP 60–200 nm**			**Presumed resting pool** > **200 nm**	
**Animal identity**	**Mean number of synaptic vesicles**	**Min–Max of synaptic vesicles**	**Mean diameter (nm)**	**(μm3)**	**(%)**	**Mean Number/ bouton**	**(%)**	**Mean number/ AZ**	**p10**	**p20**	**Mean number/ bouton**	**(%)**	**Mean number/ AZ**	**Mean number/ bouton**	**%**
R-ad 151106_I	836.75 ± 502.04	280–2047	35.51 ± 3.11	0.02 ± 0.01	7.40	61.10 ± 31.90	7.30	50.85 ± 27.65	3.10 ± 3.03	14.00 ± 9.04	172.90 ± 76.96	18.30	138.40 ± 62.96	664.10 ± 466.58	58.52
R-ad 170407_I	797.81 ± 497.92	122–1652	31.41 ± 2.26	0.01 ± 0.01	3.48	63.00 ± 41.68	7.90	53.98 ± 38.95	4.71 ± 3.54	15.10 ± 10.85	138.33 ± 98.57	18.37	114.45 ± 83.72	596.48 ± 391.41	72.10
R-ad 170407_Id	1069.66 ± 620.86	166–2630	30.93 ± 2.24	0.02 ± 0.01	5.31	74.20 ± 44.76	6.93	66.62 ± 37.94	5.29 ± 5.80	17.68 ±12.98	200.83 ± 118.25	20.56	180.20 ± 95.06	794.63 ± 505.86	71.24
R-ad 170407_II	502.40 ± 495.79	47–1069	32.18 ± 2.04	0.01 ± 0.01	5.95	39.40 ± 22.46	7.85	31.80 ± 9.88	2.40 ± 1.82	7.80 ± 1.79	112.00 ± 91.74	32.85	91.20 ± 74.75	351.00 ± 385.54	47.39
GS_R_ad 020205_I	1163.60 ± 569.93	537–1882	43.18 ± 2.32	0.06 ± 0.03	6.06	68.80 ± 23.79	5.91	68.80 ± 23.79	1.80 ± 2.49	11.40 ± 7.67	205.20 ± 65.89	19.59	205.20 ± 65.89	889.60 ± 497.87	73.51
GS_R_ad 070605_I	888.24 ± 459.41	219–2087	33.28 ± 3.89	0.02 ± 0.02	7.27	67.12 ± 30.19	7.56	60.03 ± 30.19	0.76 ± 1.09	7.35 ± 6.27	234.12 ± 113.99	29.25	211.79 ± 120.57	587.00 ± 384.48	62.18
GS_R_ad 150805_I	421.82 ± 332.33	111–1178	29.77 ± 1.71	0.01 ± 0.01	4.05	32.00 ±17.40	7.58	32.00 ± 17.40	2.91 ± 2.12	7.55 ± 5.05	76.45 ± 41.79	21.49	76.45 ± 41.79	313.36 ± 289.33	67.66
Mean	811.47 ± 272.25	–	33.75 ± 4.55	0.02 ± 0.02	5.78	57.94 ± 15.91	7.29	52.01 ± 15.13	3.89 ± 3.35	11.55 ± 4.16	162.83 ± 56.37	22.92	145.38 ± 54.61	599.45 ± 212.21	64.66
Median; IQR	836.75; 567.26	–	32.18; 4.58	0.02; 0.01	–	63.00; 29.40	–	53.98; 34.62	2.91; 3.10	11.40; 7.55	172.90; 93.20	–	138.40; 114.00	596.48; 443.63	–
CV	0.36	–	0.13	1.00	–	0.20	–	0.29	0.86	0.36	0.35	–	0.38	0.35	–
Skew	−0.32	–	1.84	1.98	–	−1.00	–	−0.53	2.07	0.32	−0.39	–	0.05	−0.16	–
Kurtosis	−0.98	–	*3.63*	*4.54*	–	−0.73	–	−1.34	*4.90*	−1.71	−1.06	–	−1.95	−1.01	–

*Summary of structural parameters relevant for release that have been extracted from the detailed 3D-reconstructions. Values are taken from conventionally embedded electron microscopic material (R-ad) and sections processed for glutamine synthetase (GS_R_ad) subsequently embedded for EM. Means ± SDs are given for individual animals. Italic numbers represent measurements with a skew or kurtosis over 3, indicating a non-normal distribution*.

Only a weak correlation was found between the total number of synaptic vesicles and the boutons surface area or volume or the PreAZs surface area (Figures 6C–E, *R*^2^ = 0.15, 0.26, 0.18, respectively), indicating that the size of the total vesicular pool is regulated independently from that of the bouton and the PreAZ.

**Figure 9:**
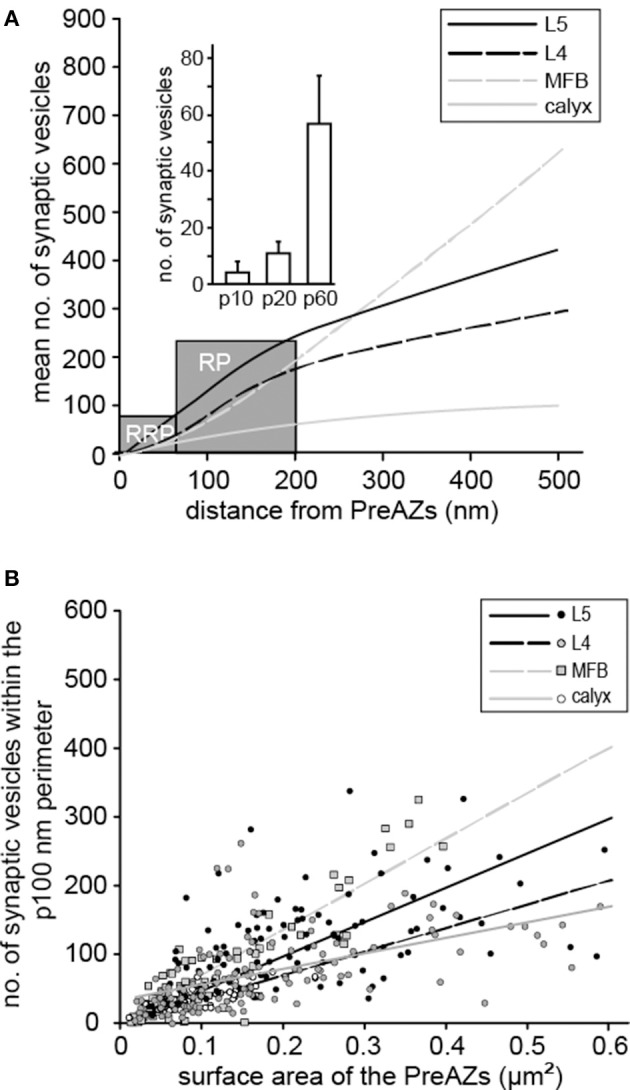
Comparison of the three pools of synaptic vesicles at different CNS synapses. **(A)** Correlation of the averaged total number of synaptic vesicles as a function of distance to the PreAZ for L5B synaptic boutons (solid black line), L4 synaptic boutons (dashed black line), MFB (dashed gray line), and the calyx of Held (solid gray line). The gray boxes indicate the borders between the calculated RRP, the RP and the resting pool of synaptic vesicles. Inset, bar histogram of the RRPs for the p10, p20, and p60 nm perimeter, respectively. **(B)** Correlation of the number of synaptic vesicles within the calculated RP at a perimeter of 100 nm with the surface area of PreAZs for L5B synaptic boutons (black dots and black solid regression line), L4 synaptic boutons (gray dots and black dashed regression line), MFBs (gray squares and gray dashed regression line) and the calyx of Held synapse (open circles and gray solid regression line).

An attempt was made to identify subsets of synaptic vesicles that could represent a structural correlate for the three functional pools of releasable quanta of transmitter. We counted vesicles located within different perimeters (p) of the PreAZ that reflect morphologically defined vesicle pools (Rizzoli and Betz, 2005). The perimeters were: 10 nm (docked vesicles) and 20 nm (membrane touching vesicles) that constitute a presumed RRP, between 60 and 200 nm (presumed RP) and beyond 200 nm (presumed resting pool) from the PreAZ (Figures 8A, **9**, Table 3). The average number of synaptic vesicles located within p10 nm of the PreAZ was 3.89 ± 3.35 most likely representing docked vesicles ready to be released with a single AP. Within p20 nm distance this number increased by nearly 3-fold (11.55 ± 4.16; Figure 9A inset, Table 3). No correlation was found for the size of the p10 RRP with the total pool of synaptic vesicles (r_s_ = 0.32; see also Figure 6F); however, the r_s_ values increased at p20 (0.42) and p60, respectively (0.61; see Figure 9 inset, Supplementary Figures 5A–D).

The structurally defined RP/bouton and the RP/AZ were also comparably large with 162.83 and 145.38 synaptic vesicles at p60–200 nm, respectively (~20% of the total pool). The structural estimate of the RP is ~2-fold larger than our functional estimate, although their high variability implies an overlap in a fraction of the synapses. The resting pool contained on average 599.45 synaptic vesicles representing ~60% of the total pool.

In summary, in L5B excitatory synaptic boutons relatively large numbers of vesicles could be assigned to the RRP and RP, based on their distance from the PreAZ. Comparison of the functional pools with vesicles distance distribution suggests that in L5B boutons, the RRP is located within 20 nm, while the RP was within 60–200 nm of the PreAZ. Although, the great variability between individual synaptic boutons implies that a greater range of geometrical distances can accommodate the functional vesicle pools.

### Two synaptic bouton classes distinguished by vesicle distributions

**Figure 10:**
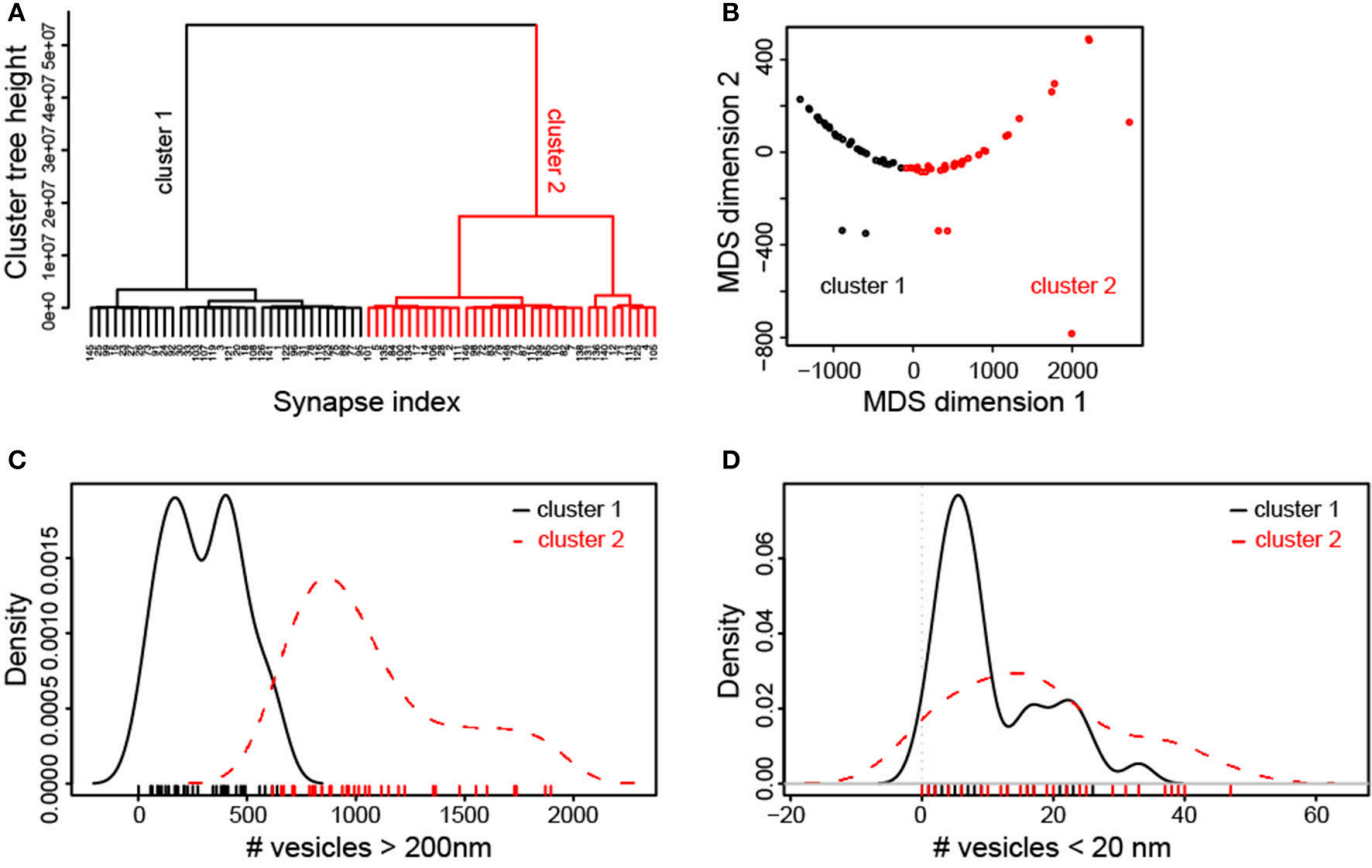
Cluster analysis of synaptic parameters of L5B excitatory synaptic boutons. **(A)** Hierarchical cluster analysis indicates two main classes of synapses. The studied synapses (index of cases on x-axis) were characterized by a range of non-overlapping synaptic parameters. As indicated by the relative length of the tree height interval, the two-cluster solution dominated across the scales of potential solutions. **(B)** Multidimensional scaling plot of synaptic parameters. This plot confirms that the two main groups of synapses that were defined by the HCA and are indicated as red and black data points are spatially non-overlapping. The diagram also indicates that the data are structured along a main linear gradient of characteristic synaptic parameters. **(C)** Density plots of the number of vesicles at distances > 200 nm from the PreAZ show strong separation of the two classes of synapses indicated by the cluster analysis. **(D)** By contrast, density plots of the number of vesicles at distances <20 nm from the PreAZ show wide overlap between the two classes of synapses.

The broad distribution of structural parameters and their relative weak correlation, in particular those of synaptic vesicles (Figure 6, Supplementary Figure 5) could be also related to the presence of several types of synaptic boutons within our large sample. To examine this possibility, we initially performed a hierarchical cluster analysis (HCA) of all structural synaptic parameters investigated (see Material and Methods). This analysis revealed two principal clusters of synaptic boutons that were stable over a large interval of the cluster tree (euclidian) height (Figure 10A). A second method of multivariate analysis, multidimensional scaling (MDS, Figure 10B) confirmed that the two clusters defined by the HCA, (color-coded by red and black data points) were not spatially overlapping. The continuous, linear arrangement of the data points in the MDS plot, however, also suggested that the data are structured by a main parameter gradient, beyond the existence of categorical clusters. Thus, the two approaches demonstrated that synaptic boutons can be grouped into two main, intrinsically graded classes by their structural features.

Next, we investigated which structural parameters most clearly separated the synaptic boutons into the two clusters. The cluster label was added to the original dataset (indicating whether individual synaptic boutons belonged to cluster 1 or 2), and density plots of each parameter were constructed for each cluster. Visual investigation of these plots as well as non-parametric statistical comparisons showed that the clusters were best separated by the total number of vesicles and by the number of vesicles located at distances >200 nm from the PreAZ (Figure 10C). In contrast, the number of vesicles closer to the PreAZ (<20 nm) was not significantly different between the clusters (Figure 10D). Interestingly, spine and shaft synapses were not distinguishable by any of the tested parameters (Supplementary Figure 6). These analyses revealed two subtypes of L5B synaptic boutons: those with a small resting pool of vesicles and the other with a large one; although both have similarly-sized RRPs and RPs.

### Glial coverage of individual synaptic complexes in L5B

Pre-embedding immunohistochemistry against glutamine synthetase, a key enzyme in astrocytes, was carried out to examine the structural relationship between individual L5B synaptic complexes and astrocytes. In addition, double immunohistochemistry for glutamine synthetase and GABA was performed, since it has been demonstrated that a subset of astrocytes in the hippocampus contained GABA instead of glutamate (LeMeur et al., 2012).

Therefore, synaptic boutons (*n* = 33), their postsynaptic target dendrites or spines and astrocytic processes were completely 3D-reconstructed and additional astrocytes (*n* = 50) were investigated with respect to whether they contain GABA gold particles.

**Figure 11:**
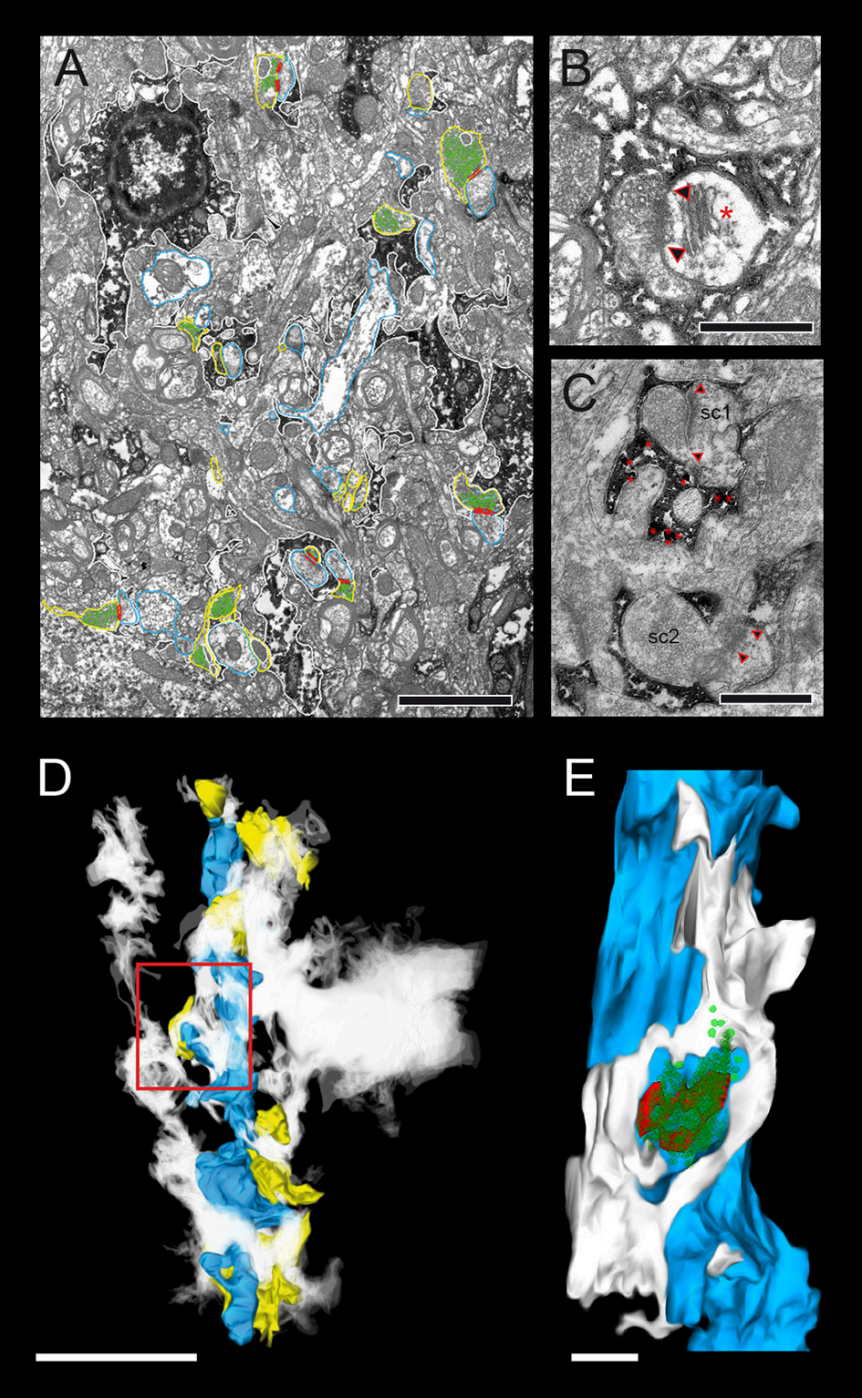
Astrocytic coverage of L5B excitatory synaptic boutons. **(A)** Low power electron micrograph showing the distribution of astrocytic processes (dark DAB-reaction product outlined in white) comprising a dense network in L5B. Here, all reconstructed synaptic boutons are outlined in yellow, dendritic segments in blue, AZs in red, synaptic vesicles in green. Scale bar in **(A)** 1 μm. **(B)** A synaptic complex composed by an *en passant* synaptic bouton on a dendritic spine is completely ensheathed by fine astrocytic processes (dark DAB-reaction product). The AZ is indicated by the arrowheads, a spine apparatus by an asterisk. **(C)** Here, immunohistochemistry against GABA was carried out identified as large silver-intensified gold grains (for better visualization marked by red asterisks) in astrocytic processes around sc1, whereas those around sc2 were immune-negative for GABA. Scale bars in **(B**,**C)** 0.5 μm. **(D)** 3D-volume reconstruction of a basal dendritic segment (blue) and several synaptic boutons (yellow) surrounded by a dense network of astrocytic processes given as a white cloud. Scale bar: 0.5 μm. **(E)** Enlargement of the framed area in **(D)** showing an individual PreAZ (red) with its pool of synaptic vesicles (green dots) tightly ensheathed by fine astrocytic processes (white) that could be followed to reach the PreAZ. Scale bar in **(E)** 0.25 μm.

In general, astrocytes and their fine processes formed a dense network in L5B (Figures 11A,D). The majority (~95%) of synaptic complexes, composed of a synaptic bouton and its postsynaptic spine or dendritic shaft were tightly ensheathed by fine astrocytic processes (Figures 11B,C,E) physically isolating the synaptic complex from the surrounding neuropil (Figure 11B) and from other adjacent synaptic complexes (Figures 11B,C). In ~30% of the astrocytes and their fine processes, large gold particles were observed, indicative for the presence of GABA (Figure 11C). These astrocytes were intermingled with the non-GABA containing population of astrocytes (Figure 11B). In both cases, fine astrocytic processes were observed reaching as far as the synaptic cleft under the PreAZ and PSD (Figures 11B,C,E) indicative for a role in the induction, maintenance and termination of synaptic transmission and in shaping the temporal and spatial glutamate concentration profile at the synaptic cleft at L5B synaptic complexes.

## Discussion

Our comprehensive functional and structural analysis of L5B excitatory synapses indicate simultaneous release of multiple synaptic vesicles from the functionally defined RRP that corresponds to a small pool of docked and short distance vesicles (p10 and p20 nm from the PreAZ). Replenishment of the RRP occurs rapidly from a larger RP located approximately within 60–200 nm of the PreAZ. Synaptic efficacy and temporal dynamics are determined by the size of the RRP and RP, respectively. These pools are independent and their heterogeneous combination give rise to the great variability in release efficacy, *P*_*r*_ and temporal dynamics. The functionally estimated RRP and RP nearly matched the structurally defined ones. An unusually large resting pool of vesicles was found in roughly half of the synaptic boutons and together with the large AZs, abundance of numerous mitochondria and dense astrocytic ensheathment support the remarkably sustained release observed during prolonged high-frequency firing at these synapses.

### Shape and size of AZs and *P_r_*

The size and shape of the AZ are important factors affecting the reliability, size of the RRP and *P*_*r*_ at individual synapses. This is the case in the hippocampus, where larger AZs are correlated with higher *P*_*r*_, larger RRPs and a higher number of presynaptic Ca^2+^ channels (Matz et al., 2010; Freche et al., 2011; Holderith et al., 2012). Perforations of the AZ are linked to enhanced synaptic efficacy (Peters and Kaiserman-Abramof, 1969; Geinisman et al., 1991; Geinisman, 1993; Nava et al., 2014). Moreover, the shape and size of AZs can be regulated in an activity-dependent manner, sometimes within minutes after stimulation (Matz et al., 2010).

At L5B excitatory synaptic boutons, the PreAZs and PSDs were on average 0.29 ± 0.19 μm^2^ and 0.31 ± 0.21 μm^2^, respectively. Strikingly, AZs were ~2 to 5-fold larger in surface area compared to other CNS synapses of similar bouton size [climbing fiber synapses 0.14 μm^2^ (Xu-Friedman et al., 2001); parallel fiber synapses of rat cerebellum 0.13 μm^2^ (Xu-Friedman et al., 2001), glutamatergic synapses in the hippocampal CA1 region 0.06 μm^2^ (Harris and Stevens, 1989; Schikorski and Stevens, 2001; Marrone et al., 2005)] or even of much larger boutons [calyx of Held in young rats 0.10 μm^2^ (Sätzler et al., 2002); endbulb of Held in rats 0.06 μm^2^ (Nicol and Walmsley, 2002); adult cats 0.14 μm^2^ (Ryugo et al., 1997); hippocampal mossy fiber boutons (MFBs) 0.11 μm^2^ (Rollenhagen et al., 2007)]. Surprisingly, albeit their unusually large AZs and the prevalence of PSD perforations (~35% of AZs), L5B excitatory synaptic boutons had on average only an intermediate *P*_*r*_, that was similar to hippocampal CA3-CA1 synapses (Holderith et al., 2012), but in contrast to the latter, did not correlate with either q or RRP size. Why then maintain these large AZs? One possible explanation may be indicated by the ability of these terminals to release multiple synaptic vesicles simultaneously by providing a large “docking area” and by their unusually large reservoir of vesicles. Both represent a potential for a much stronger form of release than observed under the resting conditions of our experiments. With this structural potential, L5B excitatory synapses could, in theory, rapidly change between modes of release without the burden of building or eliminating synaptic structures. Such changes may be required for the L5B-driven transitions between persistent intermediate-frequency firing during awake and Down-states and the transient high-frequency firing typical of Up-states (Sanchez-Vives and McCormick, 2000; Sakata and Harris, 2009; Lörincz et al., 2015).

The majority of synaptic boutons (~85%) investigated were established on dendritic spines of different types, the majority of which (~75%) contained a spine apparatus. It has been hypothesized, that spines containing this structure are more mobile (Deller et al., 2003). Spine motility may be required not only to ease the establishment of a new synaptic contact, but also contributes to its maintenance and stabilization with the presynaptic bouton.

### Quantal analysis and release mode in L5B-L5B excitatory synaptic connections

Our measurements of adult synapses between thick-tufted L5B-L5B pyramidal neurons reveal relatively small EPSP amplitudes, high CVs and F%, in close agreement with other reports on this connection (Hardingham et al., 2010; Kerr et al., 2013). In contrast, Brémaud et al. (2007) reported much larger amplitudes, lower CVs and failures for cortical synapses between unidentified L5 neurons. One possibility is that these synapses were contributed by L5 neurons located in different cortical regions, different sublayers (L5A vs. L5B) or separated by greater horizontal distances. Brémaud et al. (2007) demonstrated that cortical synapses can be well-described by simple binomial analysis and are characterized by layer-specific quantal parameters.

One aim of the current study was to explore how the number of synaptic vesicles and their spatial distribution relates to the probability and efficacy of release. These parameters were estimated by fitting the experimental data to the binomial quantal model of transmitter release, constrained by direct measurements of vesicular quanta (mEPSPs) and contact numbers obtained from 3D-reconstructions. This analysis generated two major conclusions: (1) Multiple vesicles are released from individual AZs during a single AP. (2) The number of simultaneously released vesicles may affect synaptic efficacy (q) independently of *P*_*r*_.

In line with our findings multivesicular release (MVR) had been demonstrated in a number of CNS synaptic connections (reviewed by Rudolph et al., 2015). Interestingly, among excitatory synaptic connections of the neocortex, MVR might occur in some but not all layers, as previous studies indicated univesicular release in L2-L2 synapses (Silver et al., 2003; Hardingham et al., 2010; Molnar et al., 2016). From the functional analysis, we estimated that 1–7 vesicles could be released simultaneously at an individual AZ upon a single AP that corresponds closely to the anatomically reconstructed pool of vesicles residing within 10 nm of the PreAZ (~1–5, average 3.89 ± 3.35). In hippocampal synapses a similar number of docked vesicles was observed that was positively correlated with the AZ surface area and *P*_*r*_ (hippocampus: Harris and Sultan, 1995; Murthy et al., 1997; Schikorski and Stevens, 1997; Shepherd and Harris, 1998; Branco et al., 2010; Holderith et al., 2012). Surprisingly, in our study neither bouton size nor AZ surface area correlated with the number of docked (10 nm) or more proximal located vesicles (<60 nm). In parallel, functional analysis suggested only a poor correlation between *P*_*r*_ and the number of vesicles released per single AP. These results could be explained if vesicular docking and release are not limited by the area of the AZ, but instead by release-competent factors in the AZ or for the “priming” of vesicles. The unusually large area of PreAZs in L5B excitatory synaptic boutons could enable such a scenario and partially account for the difference to the smaller hippocampal PreAZs. Functionally, MVR extends the range of synaptic activity and plasticity and may be important for synchronizing L5B activity thereby enhancing its output to long- and short-range cortical or subcortical targets.

### Vesicle pools in L5B-L5B synapses

The total pool size of synaptic vesicles at L5B excitatory synaptic boutons was ~750/AZ and comparable to values for rat L4 synaptic boutons (~550/AZ; Rollenhagen et al., 2015). At the adult MFB the total pool size/AZ was ~850 (Rollenhagen et al., 2007), in cerebellar MFBs an average number of ~300/AZ (Saviane and Silver, 2006) at the calyx of Held giant endterminal ~125 synaptic vesicles /AZ were observed (Sätzler et al., 2002). Although small (~20-fold smaller in volume when compared with the hippocampal MFB), those in L5B are comparable in their total pool size/AZ with even much larger CNS synapses (hippocampal and cerebellar MFBs) or even contain a larger total pool of synaptic vesicles/AZ (calyx of Held).

Synaptic transmission is carried by vesicles of the RRP and RP, which, together, generate the temporal response of the synapse. Their size and refilling rates define the functional bandwidth of the synapse. Our functional estimates of the RRP and RP yielded an average of 5.4 and 74 vesicles per bouton, respectively. These were based on the simplifying assumption that vesicle depletion dominated EPSC depression during high-frequency trains. However, receptor desensitization could also contribute to depression, resulting in an underestimate of the pool size. In contrast, re-filling of the pools through endocytosis could counterbalance depression and invoke an overestimate of the pool. The high abundance of AMPA receptors at L5B PSDs (Rollenhagen et al., 2012) and endocytosis rates greater than 0.5 s (Delvendahl et al., 2016) suggest only a negligible error in the RRP estimate, but a more substantial one for the RP. We, thus, deem our RP estimates to be closer to the lower limit of the true size. Our RRP estimate is similar to that reported in hippocampal CA1 synapses (Dobrunz and Stevens, 1997; Murthy et al., 1997), but is higher than in the giant calyx of Held endterminal (1–2 vesicles per AZ, Schneggenburger et al., 1999; Sätzler et al., 2002) or vestibular brain stem synapses (~2 vesicles, McElvain et al., 2015). In contrast, hippocampal and cerebellar MFBs contain a much larger RRP composed of 40 vesicles (Hallermann et al., 2003; Suyama et al., 2007) and 300 (Saviane and Silver, 2006).

The RP in L5B-L5B excitatory synapses is 2 to 3.5-fold larger as in cultured and native hippocampal CA3-CA1 synapses (30–45 vesicles; Harata et al., 2001a,b; Marra et al., 2012) and in the calyx of Held endterminal, when measured during afferent stimulation (~45 vesicles, de Lange et al., 2003). However, the RP is considerably smaller by 2.5 to 5.4-fold than in hippocampal (~400, Hallermann et al., 2003; Rollenhagen et al., 2007; Suyama et al., 2007) and cerebellar MFBs (Saviane and Silver, 2006). The resting vesicular pool can be estimated by subtracting the RRP and RP from the morphologically determined total vesicular pool and is larger in L5B excitatory synaptic boutons compared with CA3-CA1 synapses, MFBs and calyx of Held endterminal. The combination of intermediate-sized RRPs and RPs and very large resting pools appears unique to L5B excitatory synaptic boutons and may serve the need to respond efficiently both to short high-frequency and persistent low-frequency activity. What is even more striking is the finding that each L5B-L5B connection expressed its own specific combination of pools. This heterogeneity could reflect functional sub-circuits or “activity-units” within L5B tuned to support different activity patterns and might in itself result from the activation and plasticity history of each synapse.

### Spatial distribution of functional vesicle pools

How does the geometrical distribution of vesicles in L5B excitatory boutons map onto functional pools? In the Drosophila neuromuscular junction, the RRP, RP and resting pools are located at gradually increasing distances from the AZ (Kuromi and Kidokoro, 1998). If a similar scenario exists in L5B excitatory synaptic boutons, then vesicles located within 60 nm, between 60–200 nm and farther than 200 nm from the PreAZ, would represent the RRP, RP and resting pools, respectively. However, in most synapses investigated to date, a more complex scenario was found in which vesicles of the RRP are concentrated at the vicinity of the AZ (Imig et al., 2014), although not all vesicles close to the AZ are fully release-competent. Recycling and release-resilient vesicles are dispersed and intermingled throughout the bouton (reviewed by Denker and Rizzoli, 2010; Alabi and Tsien, 2012; Fowler and Staras, 2015), although a relative bias of the RP vesicles toward the AZ has been reported (Marra et al., 2012). Drawing on the latter scenario, we suggest a modified geometrical distribution of functional vesicular pools in L5B terminals: the RRP may be located within 20 nm of the PreAZ, containing on average ~12 vesicles, of which 30–50% would be release-competent corresponding to the ~5 and ~4 vesicles calculated from the functional analysis. The geometrical borders of the RP could be roughly delineated from the RRP-replenishment and RP-depletion rates assuming vesicle mobilization rates of 50 nm s^−1^ (Rizzoli and Betz, 2005). The lower limit of this range would be defined by the fast replenishment of the RRP within 100 ms during 10 Hz trains, corresponding to a minimal distance for the RP vesicles of 5 nm away from the RRP. The upper limit of this range is given by the complete depletion of the RP within roughly 3 s of stimulation (155 APs at 50 Hz, Figures 3D–E), which translates into ~150 nm distance. The number of vesicles located <150 nm from the L5B PreAZ is roughly 200 (Figure 9) and thereby larger than two times our RP estimate, possibly reflecting partial co-localization of RP and resting vesicle pools. Based on the same vesicle mobilization rate, we also infer that the large number of vesicles we reconstructed at distances >200 nm from the PreAZ do not contribute to the high-frequency EPSCs measured (lasting <0.5 s). These vesicles, may, however, participate in release during lower-frequency or prolonged stimulation.

Taken together, the comparably large RRP at L5B excitatory synaptic boutons paired with moderate *P*_*r*_, may prevent depletion during repetitive high-frequency stimulation and, even more importantly, the large size of the RP and resting pool may be used to rapidly refill the releasable pool if it has been depleted by long-lasting repetitive stimulation. If the refilling rates were activity dependent, the large size of the RP could explain synaptic plasticity, for instance, a substantial increase in synaptic strength during frequency facilitation and post-tetanic potentiation.

### Other structural subelements important in synaptic transmission

#### Mitochondria

L5B excitatory synaptic boutons contained a comparably high number of mitochondria that were always associated with the pool of synaptic vesicles. It has been demonstrated in the CNS that beside other functions they act as internal calcium stores regulating and adjusting internal Ca^2+^-levels in the CNS (Pozzan et al., 2000; Rizzuto et al., 2000), are highly mobile and associated with the pool of synaptic vesicles (Mironov, 2006; Mironov and Symonchuk, 2006) and even more importantly, are required for the mobilization of synaptic vesicles from the resting pool (Verstreken et al., 2005). The high abundance of mitochondria in ~97% of L5B excitatory synaptic boutons (~15% to the total volume of the nerve terminal) together with the close association with the large pool of synaptic vesicles suggest an important role for mitochondria in several of the signal cascades underlying synaptic transmission, efficacy and strength.

#### Glial coverage of L5B synaptic complexes

The majority of synaptic complexes (~85%) in rat L5B were tightly enwrapped by fine astrocytic processes reaching as far as the synaptic cleft in line with findings at other small-sized CNS synapses (Xu-Friedman et al., 2001; Rollenhagen et al., 2015), but in marked contrast to the hippocampal MFB (Rollenhagen et al., 2007), the calyx of Held-principal neuron synapse (Müller et al., 2009) and hippocampal CA1 synapses (Ventura and Harris, 1999). There, only ~50% were directly found at the synaptic interface (Ventura and Harris, 1999) suggesting that hippocampal astrocytes do not uniformly sample glutamate. At hippocampal MFBs and the calyx of Held fine astrocytic processes were never seen to reach as far as the synaptic cleft implicating glutamate spillover and as a consequence synaptic cross talk at these synapses. Astrocytes at L5B synaptic complexes thus act as physical barriers to neurotransmitter diffusion thereby preventing spillover of released glutamate by active take-up and removal of glutamate. They terminate synaptic transmission and may thus speed-up the recovery from receptor desensitization (Danbolt, 2001; Oliet et al., 2004). Both mechanisms allow the precise spatial and temporal regulation of the neurotransmitter concentration in the synaptic cleft (Anderson and Swanson, 2000).

Furthermore, astrocytes release glutamate or GABA (LeMeur et al., 2012) through vesicular exocytosis, which can also regulate synaptic transmission through activation of pre- and postsynaptic receptors (Haydon and Carmignoto, 2006). In addition, synapses with a higher number of docked vesicles as shown for L5B excitatory synaptic boutons are almost exclusively ensheathed by astrocytic processes that was correlated with higher synaptic activity (Brückner et al., 1993). Finally, astrocytes are crucial for the induction and control of spike-time dependent depression (t-LTD) at neocortical synapses by gradually increasing their Ca^2+^ signaling during the induction of t-LTD (Min and Nevian, 2012). Thus astrocytes may act as a memory buffer for previous coincident neuronal activity and therefore seem to be involved in modulating synaptic transmission and plasticity by temporal and spatial modulation of the glutamate concentration profile at L5B excitatory synaptic boutons.

## Author contributions

AR and JL performed all structural experiments, electron microscopic data acquisition and analysis, and the conception and writing of the paper. OO performed all electrophysiological experiments, analysis of the functional data and conception and writing of the paper. KS provided the software tool OpenCAR and was involved in the final analysis of the structural data. CH performed the multivariate analyses. DK provided financial support to OO and participated in writing of the paper.

### Conflict of interest statement

The authors declare that the research was conducted in the absence of any commercial or financial relationships that could be construed as a potential conflict of interest.
