# Supplementary figures and images for: Structural Properties of Synaptic Transmission and Temporal Dynamics at Excitatory Layer 5B Synapses in the Adult Rat Somatosensory Cortex
Source: Front Synaptic Neurosci. 2018 Jul 30;10:24. doi: 10.3389/fnsyn.2018.00024 (PMC6077225; doi:10.3389/fnsyn.2018.00024)

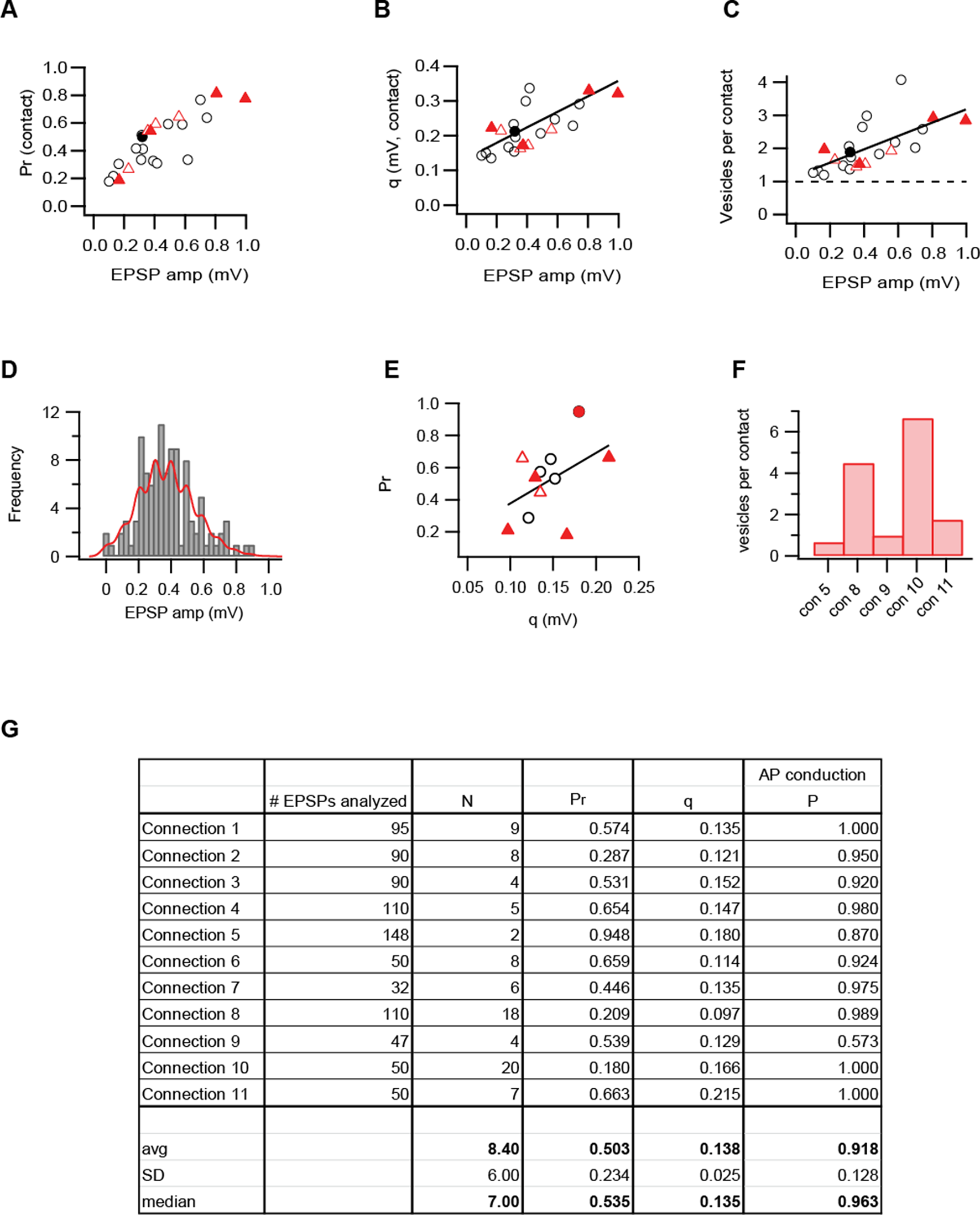

Supplement: Supplementary file 1 [file Image_1.TIF]

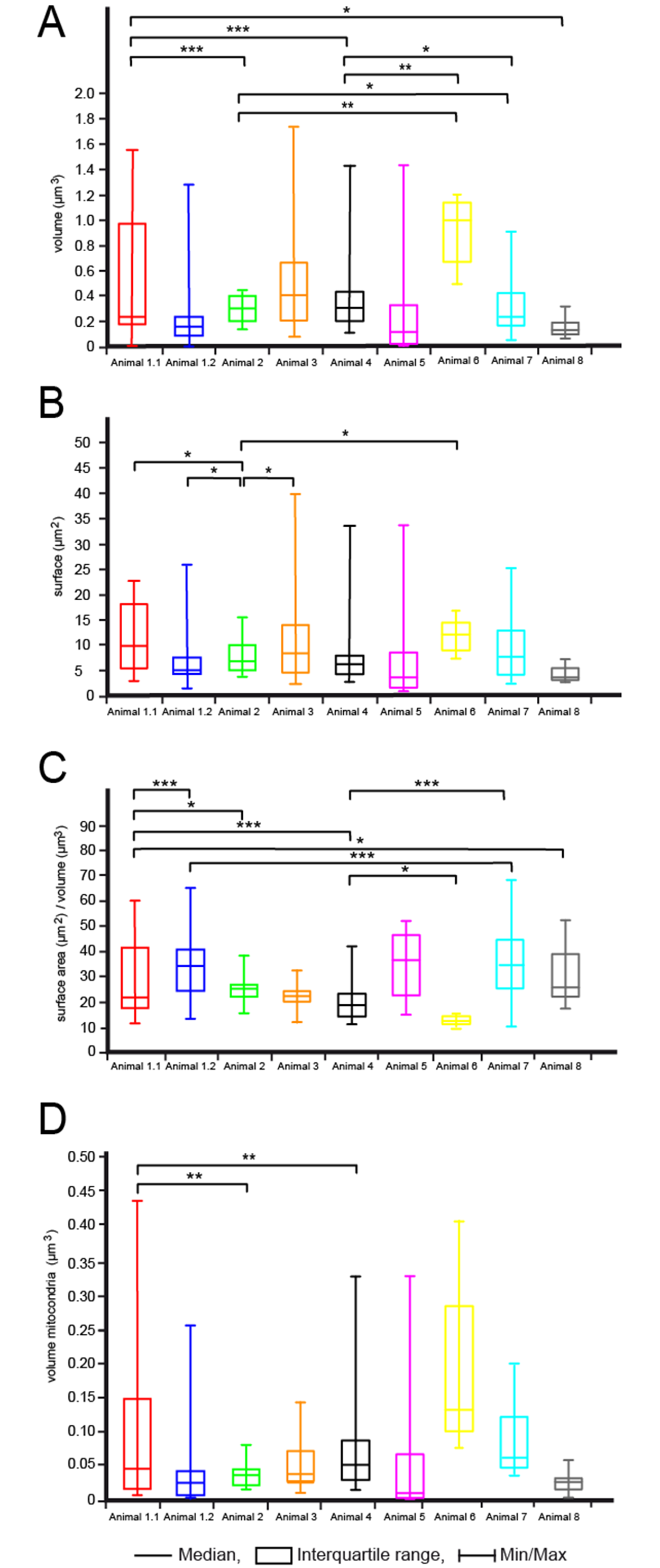

Supplement: Supplementary file 2 [file Image_2.TIF]

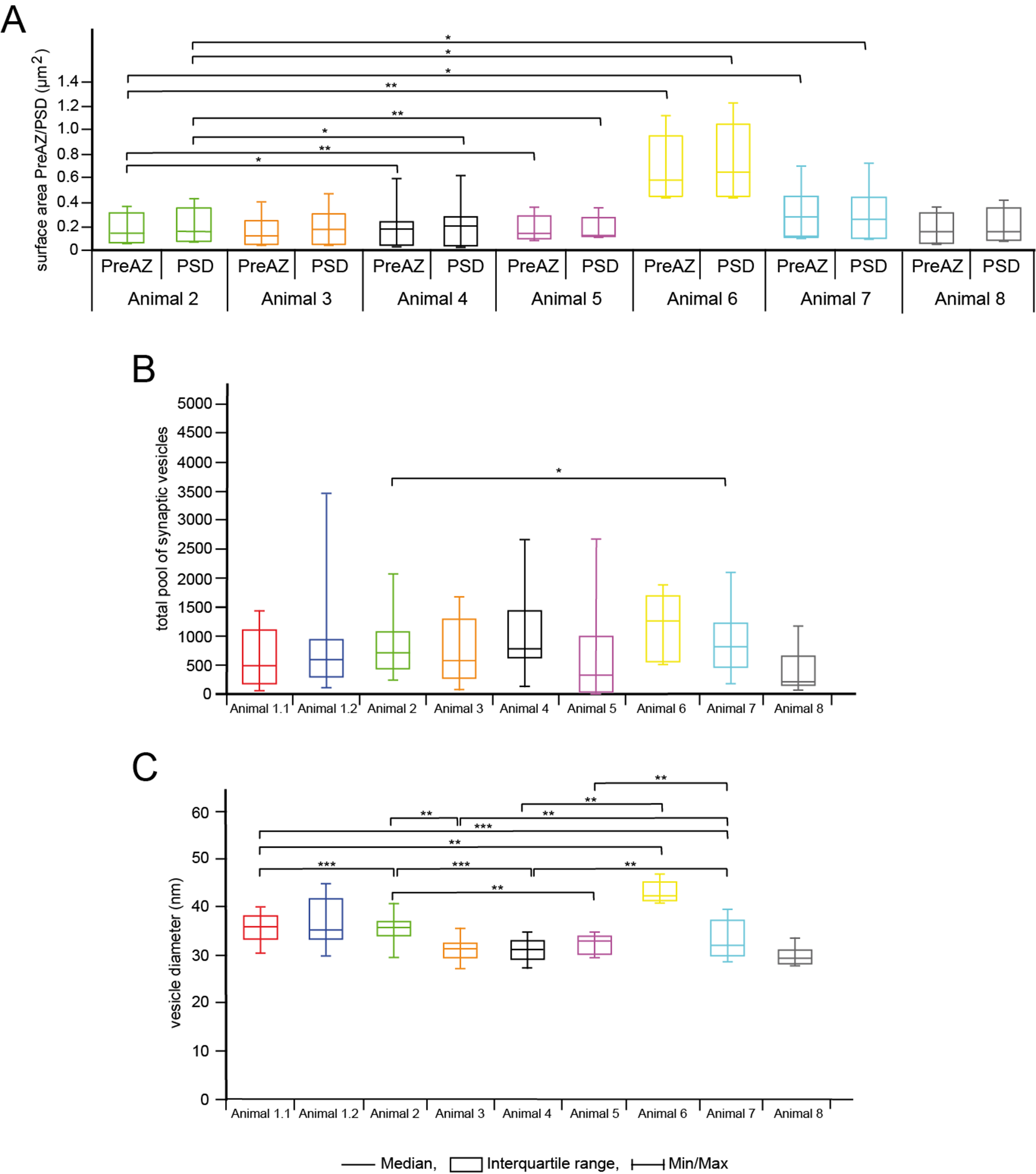

Supplement: Supplementary file 3 [file Image_3.TIF]

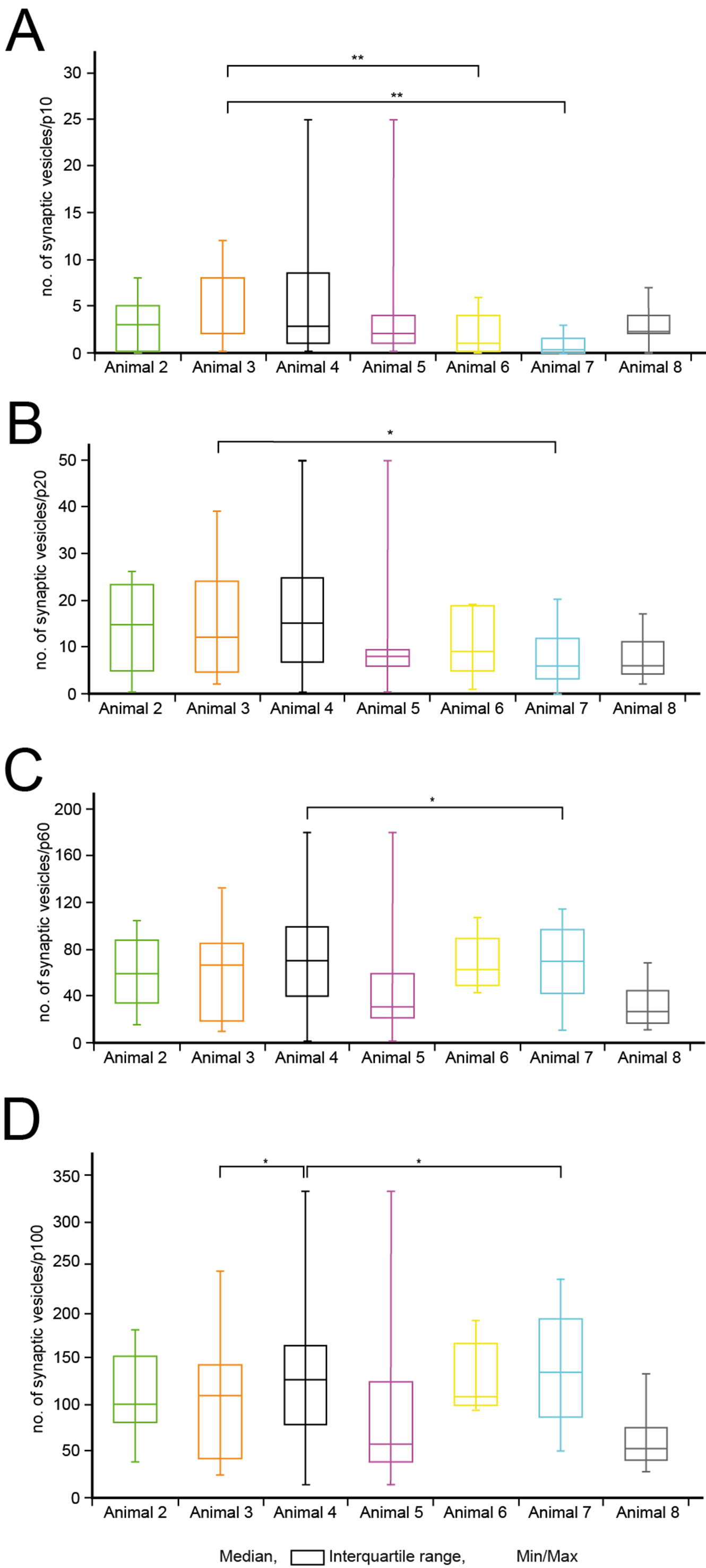

Supplement: Supplementary file 4 [file Image_4.TIF]

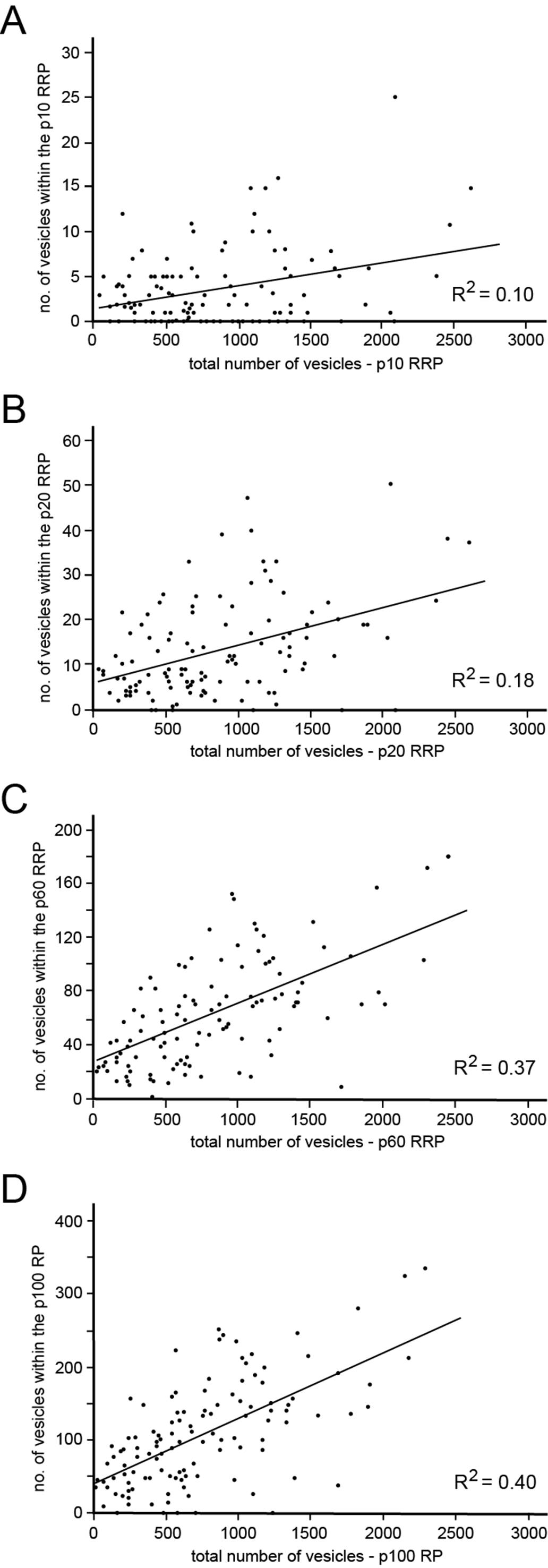

Supplement: Supplementary file 5 [file Image_5.TIF]

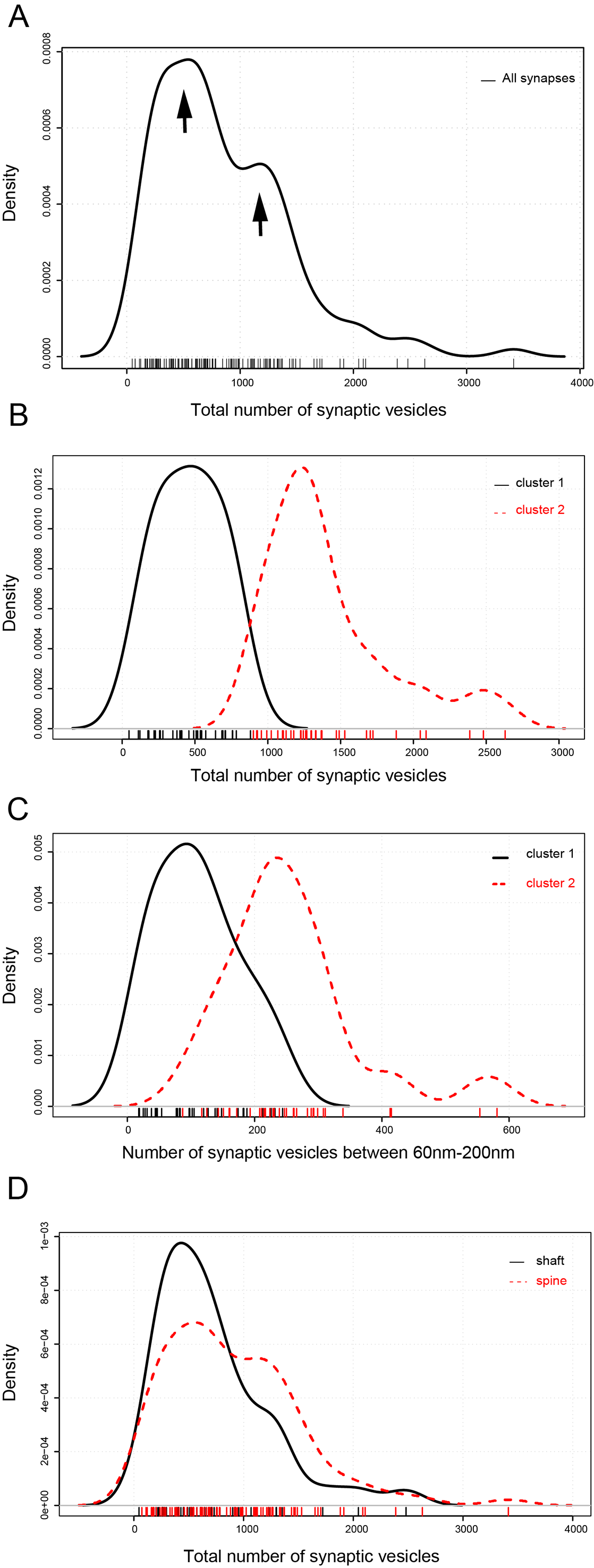

Supplement: Supplementary file 6 [file Image_6.TIF]
