# Supplementary material for: Structural Properties of Synaptic Transmission and Temporal Dynamics at Excitatory Layer 5B Synapses in the Adult Rat Somatosensory Cortex
Source: Front Synaptic Neurosci. 2018 Jul 30;10:24. doi: 10.3389/fnsyn.2018.00024 (PMC6077225; doi:10.3389/fnsyn.2018.00024)
Supplement: Supplementary file 7 [file Table_1.docx]

Supplementary Material

Structural properties of synaptic transmission and temporal dynamics at excitatory layer 5B synapses in the adult rat somatosensory cortex

Astrid Rollenhagen, Ora Ohana, Kurt Sätzler, Claus C. Hilgetag, Dietmar Kuhl

and Joachim H.R. Lübke

Corresponding Authors: Joachim HR Lübke, e-mail: [j.luebke@fz-juelich.de](mailto:j.luebke@fz-juelich.de)

and Ora Ohana, e-mail: [ora.ohana@zmnh.uni-hamburg.de](mailto:ora.ohana@zmnh.uni-hamburg.de)

**Suppl. Fig. 1: Quantal analysis of EPSP amplitudes**

**A-C**, Quantal binomial calculation of *P_r_* and q from the CV, based on anatomically-determined N of synaptic contacts. Both *P_r_* (**A**) and q (**B**) contribute to the EPSP amplitude. Filled symbols represent connections for which the number of synaptic contacts was determined from the 3D- reconstructions.

**C**, The number of vesicles per contact was calculated by dividing q by q_vesicle_ and co-regulates with the EPSP amplitude. Dashed line marks a single vesicle released.

**D-G**, Binomial quantal fits of the EPSP amplitude histogram provides an unbiased estimate of *P_r_*, q and N in each connection.

**D**, Example of an EPSP amplitude from one connection (connection 8 in the table) fitted with the models probability density function (red line). The model predictions were: N=18, *P_r_*=0.209 mV and q=0.097 mV.

**E**, Non-significant correlation between *P_r_* and q (r^2^=0.208, P=0.15, two-tailed test). Symbols as in A-C.

**F**, Predicted N from the model was assumed to reflect the number of vesicles in the connection and was divided by the number of anatomically-determined contacts. Number of vesicles per contact is displayed for each of the reconstructed connections.

**G**, Table of the quantal estimates of the model for 11 connections in which a satisfactory fit could be obtained.

**Suppl. Fig. 2: Box plots of various synaptic parameters comparing individual animals**

Data distributions as indicated by the medians (horizontal bars) and interquartile ranges (framed areas) and minimum and maximum (vertical lines) are shown for volume (A), surface area (B), surface vs. volume (C) and volume of mitochondria (D). Asterisks indicate significant differences (*: p<0.05; **: p<0.01; ***: p<0.001).

**Suppl. Fig. 3: Box plots of various synaptic parameters**

Data distributions as indicated by the medians (horizontal bars) and interquartile ranges (framed areas) and minimum and maximum (vertical lines) are shown for the surface area of PreAZs and PSDs (A), the total pool of synaptic vesicles (B), and vesicle diameter (C). Asterisks indicate significant differences (*: p<0.05; **: p<0.01; ***: p<0.001).

**Suppl. Fig. 4: Box plots of the RRP and RP**

Data distributions as indicated by the medians (horizontal bars) and interquartile ranges (framed areas) and minimum and maximum (vertical lines) are shown for the number of synaptic vesicles within a perimeter of 10 nm from the PreAZ (A), for the 20 nm perimeter (B), for the 60 nm perimeter (C) and the 100 nm perimeter (D). Asterisks indicate significant differences (*: p<0.05; **: p<0.01; ***: p<0.001).

**Suppl. Fig. 5: Correlations of the RRPs and RPs with the total pool of synaptic vesicles**

for the p10 RRP (A), for the p20 RRP (B), for the p60 perimeter (presumed RP) (C) and for the p100 RP (D). Data are fitted by linear regression. Note that the R^2^-values steadily increase with increasing perimeter distance.

**Suppl. Fig. 6: Cluster analysis of L5B synaptic boutons**

**A**, Density plot of the total number of vesicles in all synapses showing two prominent peaks (indicated by arrows) strongly deviating from a single Gaussian distribution.

**B**, Density plots of the total number of vesicles showing strong divergence of the two classes of synapses identified by the cluster analysis.

**C**, Separation of the two clusters of synapses becomes first significant in terms of vesicles located 60-200 nm from the PreAZ, whereas the two classes cannot be distinguished in terms of vesicles closer to the PreAZ.

**D**, Spine and shaft synapses onto L5B dendrites are not distinguishable by their structural parameters. Density plots of the total number of vesicles at spines and shafts largely overlap and are thus not distinguishable by their structural parameters.

| parameter | Median (class_1) | Median (class_2) | chi-squared test statistics | p-value  (uncorrected) |
| --- | --- | --- | --- | --- |
| Bouton area | 5.203380 | 9.992521 | 15.775 | **7.133e-05** |
| Nr. mitochondria | 1 | 1 | 6.0194 | 0.01415 (ns after Bonferoni correction) |
| Mitochondria  % volume | 17.08325 | 13.06276 | 5.7465 | 0.01652 (ns after Bonferoni correction) |
| Mitochondria volume | 0.03444394 | 0.06368998 | 6.1476 | 0.01316 (ns after Bonferoni correction) |
| AZ area | 0.1549354 | 0.3266312 | 16.151 | **5.849e-05** |
| PSD area | 0.1464064 | 0.2985069 | 18.195 | **1.994e-05** |
| Nr. AZ/ bouton | 1 | 1 | 3.2634 | 0.07084 (ns after Bonferoni correction) |
| bves.diam.mean | 30.56003 | 32.17545 | 2.2978 | 0.1296 (ns after Bonferoni correction) |
| Vesicles (total) | 474.5 | 1282.5 | 51.718 | **6.406e-13** |
| Vesicles percent volume | 3.117127 | 5.735273 | 17.896 | **2.334e-05** |
| Vesicles volume | 0.008417447 | 0.023680158 | 44.863 | **2.113e-11** |
| Bouton volume | 0.2211397 | 0.4915824 | 22.873 | **1.73e-06** |
| Ves.p10 | 2.0 | 3.5 | 2.1761 | 0.1402 (ns after Bonferoni correction) |
| Ves.p20 | 7 | 16 | 5.3169 | 0.02112 (ns after Bonferoni correction) |
| Ves.p60 | 44 | 77 | 17.504 | **2.867e-05** |
| Ves.p100 | 76.0 | 147.5 | 25.954 | **3.497e-07** |
| Ves.p200 | 147 | 318 | 32.818 | **1.012e-08** |
| Ves.p500 | 195.0 | 600.5 | 37.627 | **8.566e-10** |
| Ves. In 60-200 nm | 102 | 235 | 33.158 | **8.497e-09** |
| Ves>200nm | 309 | 966 | 51.384 | **7.597e-13** |

**Suppl. Table 1: Cluster analysis of structural synaptic parameters.**

_Assessment of the segregation of structural parameters for the two principal clusters of synapses. Differences between the clusters were assessed by the non-parametric Kruskal-Wallis rank sum test (uncorrected p-values). P-values which survived the Bonferoni multiple-comparisons corrections were marked in bold._
